# Phylogeography of the Italian vairone (*Telestes muticellus*, Bonaparte 1837) inferred by microsatellite markers: evolutionary history of a freshwater fish species with a restricted and fragmented distribution

**DOI:** 10.1186/1471-2148-10-111

**Published:** 2010-04-27

**Authors:** Flavio Marchetto, Serena Zaccara, Frauke M Muenzel, Walter Salzburger

**Affiliations:** 1 Department of Biotechnology and Molecular Sciences, University of Insubria, via Dunant 3, 21100 Varese, Italy; 2 Zoological Institute, University of Basel, Vesalgasse 1, 4055 Basel, Switzerland

## Abstract

**Background:**

Owing to its independence from the main Central European drainage systems, the Italian freshwater fauna is characterized by a high degree of endemicity. Three main ichthyogeographic districts have been proposed in Italy. Yet, the validity of these regions has not been confirmed by phylogenetic and population genetic analyses and a phylogeographic scenario for Italy's primary freshwater fish fauna is still lacking. Here, we investigate the phylogeography of the Italian vairone (*Telestes muticellus*).

**Results:**

We sampled 38 populations representing the species' entire distribution range and covering all relevant drainage systems, and genotyped 509 individuals at eight variable microsatellite loci. Applying various population genetic analyses, we identify five distinct groups of populations that are only partly in agreement with the proposed ichthyogeographic districts. Our group I, which is formed by specimens from Veneto and the Po River system draining into the Adriatic Sea, corresponds to the Padano-Venetian ichthyogeographic district (PV), except for two Middle Adriatic drainages, which we identify as a separate group (III). The Tuscano-Latium district (TL) is equivalent to our group V. A more complex picture emerges for the Ligurian drainages: populations from Central Liguria belong to group I, while populations from West (group II) and East Liguria (group IV) form their own groups, albeit with affinities to PV and TL, respectively.

**Conclusions:**

We propose a phylogeographic scenario for *T. muticellus *in which an initial *T. muticellus *stock became isolated from the 'Alpine' clade and survived the various glaciation cycles in several refugia. These were situated in the Upper Adriatic (groups I and II), the Middle Adriatic (group III), (East) Liguria (group IV) and Tuscano-Latium (group V). The population structure in the vairone is, in principal, in agreement with the two main ichthyogeographic districts (PV and TL), except for the two populations in the Middle Adriatic, which we identify as additional major "district".

## Background

It is now well established that the Quaternary ice ages were among the main contributing factors shaping the present distribution of species and of populations within species [1-3]. At least in the temperate and polar zones, the cyclic climate changes caused a shift of biomes in a North-South direction and repeatedly produced the situation that different populations belonging to the same species were separated by geographic barriers [4]. Species inhabiting North America and Europe, the two continents that were covered by the most extensive glacial sheets, were influenced in different ways with regard to the impact of ice ages on migration: in North America the main geographical barriers had the same orientation as the biomes' shift, while in Europe, due its complex geography, strong barriers to migration existed. The Mediterranean Sea, for example, limited migration towards North Africa so that many taxa were restricted to the large southern European peninsulas - the Iberian, the Balkan and the Italian - during the maximal glacial expansions. In addition, the most important mountain chains bordering these glacial refugia limited areal re-expansion towards Central and northern Europe during the interglacial periods. The combination of these two geographical features isolated populations of various taxa, thereby triggering genetic diversification and (allopatric) speciation [2].

Phylogeography, *i.e. *the integration of the phylogenetic inferences and/or population genetic relationships among individuals or taxa on one hand, and their geographic distribution on the other hand [5], offers the means to reconstruct the historical processes that triggered the geographic distribution of biodiversity. In this context, freshwater fishes constitute an important model group because their dispersion, diversification and speciation are strictly related to the processes that shaped the landmass and the hydrogeographic systems, which they inhabit [6,7]. There are only few ways by which a primary freshwater fish can reach a new river basin: by river capture [8], by downstream river confluence when the sea level is lowered [9], by dispersal over the sea under lower salinity conditions ("lago-mare" phase; [10]), or by lakes formed during the retreat of glaciers [6].

**Figure 1:**
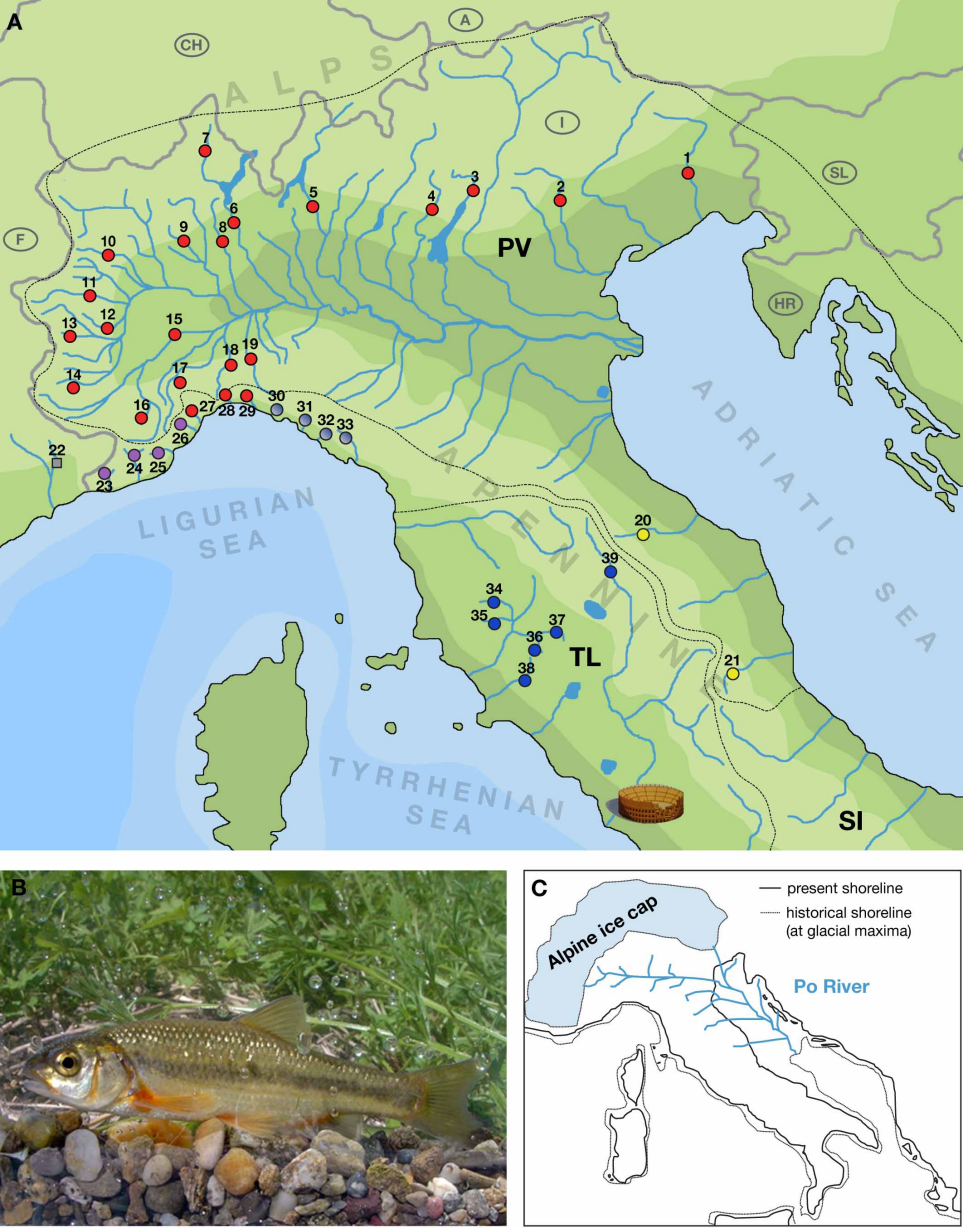
**The vairone in Italy**. (*a*) Map of northern and Central Italy showing the main river systems, the ichthyogeographic districts and the sampling sites. Numbers (and colors) of the sampling sites correspond to Table 1 and Fig. 2. Note that population 22 consisted of specimens of *Telestes souffia*, the sister species to *T. muticellus*, and was excluded from some of the analyses. (*b*) The Italian vairone (*Telestes muticellus*). (*c*) During glacial maxima, the Alps were covered by an ice cap and sea levels were markedly lowered; the estuary of the Po River system, which also included rivers that today directly drain into the Adriatic Sea, was situated at the Middle Adriatic pitch. PV...Padano-Venetian ichthyogeographic district; TL...Tuscano-Latium ichthyogeographic district; SI...South Italian ichthyogeographic district.

The Italian peninsula - isolated from continental Europe by the Alps, the highest mountain chain with an East-West orientation - hosts a relatively high number of endemic freshwater taxa [10-13]. Its independence from the main Central European drainage systems Danube, Rhine and Rhône favored this high degree of endemicity. The Italian hydrographic structure is largely influenced by the North-South orientated Apennine barrier, which modulated the allopatric distribution and the diversification of various freshwater taxa [10]. Based on the distribution of cyprinid fishes, three main ichthyogeographic districts were identified [10]: (*i*) the Padano-Venetian district (PV) including basins from the Vomano river to the Krka river, which all drain into the Adriatic Sea; (*ii*) the Tuscano-Latium district (TL) from the Serchio river to the Tevere river, which drain into the middle Tyrrhenian Sea; and (*iii*) the South Italy district (SI) (Fig. 1). Despite the importance of the Italian peninsula as major glacial refugia in general and its role in shaping the distribution and diversity of the Central European freshwater fish fauna in particular, little is known about the phylogeography of freshwater fishes in that area, and detailed studies based on molecular markers and including specimens from all relevant river basins are lacking. Therefore, limited knowledge on the recent evolution of the Italian hydrographic systems at regional and local scale is available, and a clear-cut picture of the more recent changes in the Italian hydrographic structure is lacking.

The vairone is a primary freshwater fish belonging to the family Cyprinidae, it is distributed in and around the Alps, and represents a good candidate for phylogeographic inference [14-17]. The taxonomic status and the generic assignment of the vairone are, however, under debate. Some authors stick to its original genus name *Leuciscus *[14-16], while others assign it to *Telestes *based on its position in the phylogeny of European cyprinids [18-21]. Phylogenetic analyses based on mitochondrial DNA (mtDNA) revealed the existence of two main lineages within the vairone, *T. souffia *and *T. muticellus *(note that *T. souffia *is listed as subspecies of *Telestes souffia *by some authors [14,15], while being granted species rank by others [16,17,21]). In the present study, we use the latter assignment, *i.e. Telestes muticellus*, because of its great genetic distance (> 5% in mtDNA) to the 'Alpine' clade [15].

The vairone inhabits cold running waters with high levels of dissolved oxygen. Its diet primarily consists of benthic invertebrates and, occasionally, of algae and plant remains. It exhibits a gregarious behavior, and the breeding period lasts from May to August; egg deposition takes place in the nocturnal hours on gravel, and larvae hatch after six days. Sexual maturity is reached after two to three years. The vairone is included in the red list of threatened species by the International Union for Conservation of Nature (IUCN) [22] as "Least Concern", and is listed in the Appendix III of the Bern Convention. In the last years, the Italian vairone has suffered a decrease in distribution range and in population size due to habitat degradation.

Here, we use the endemic Italian vairone (*Telestes muticellus*) as a model to assess the phylogeographic history of primary freshwater fishes in Italy [23]. This species appears highly suitable for this purpose, as it has a fragmented distribution that is restricted to Central and northern Italy and, there, covers the two main ichthyogeographic districts (PV and TL). Previous phylogeographic studies based on mtDNA indicated a population genetic structure largely in agreement with these ichthyogeographic districts but, at the same time, highlighted a more complex distribution of haplotypes in the Tyrrhenian basins of Liguria, where the freshwater fauna is considered introduced [10,24,17]. However, these studies were based on relatively small sets of populations only, and the results have not been corroborated with nuclear DNA markers. In this study, we used a sample of 38 populations (N = 509) covering the entire distribution range of *T. muticellus *and representing all relevant drainage systems, and applied eight microsatellite markers to infer the phylogeographic history of this species. Specifically, we were interested in the question whether populations of *T. muticellus *are structured according to the main ichthyogeographic districts that have been proposed on the basis of the distribution of cyprinid fish but without taking genetic information into account. We also aimed at identifying potential glacial refugia permitting vairone populations to outlive periods of maximal glacial extensions during Quaternary ice ages, as well as possible post-glacial re-colonization routes. The "permeability" of major barriers (such as the Apennine) was of particular interest to us. Finally, by integrating our population genetic data, sea level fluctuations information and the bathymetric profiles of the Adriatic Sea and the Tyrrhenian Sea, we intended to reconstruct the ways by which vairone populations dispersed and to suggest the recent changes in the hydrographic pattern that allowed the dispersion of the vairone from one basin into another.

## Methods

### Sampling

A total of 509 individuals from 38 populations were sampled between 2002 and 2008 covering the entire distribution range of *T. muticellus *(Fig. 1, Table 1). We sampled between four and twenty individuals per population so that each of the drainage systems was represented by at least 20 individuals. Twenty-one populations belong to the Padano-Venetian ichthyogeographic district (pops. 1 - 21), six to the Tuscano-Latium district (pops. 34 - 39) and eleven to Ligurian basins (pops. 23 - 33) draining into the Tyrrhenian Sea. A single population of 15 individuals belonging to the subspecies *T. souffia souffia *from South-East France (pop. 22; Fig. 1, Table 1) was included in order to evaluate possible hybridization events with neighboring populations of *T. muticellus *(see [14,15]). All specimens were collected using electric fishing gear; a clip of the anal fin was preserved in 100% ethanol and stored at 4°C as DNA sample.

**Table 1:** Sampling locations of the *T. muticellus *(and *T. souffia*; pop. 22) populations analyzed in this study.

Population	River	River basin	n° individuals	District	Drainage	GPS coordinates	
1	Tagliamento	Tagliamento	14	A	Venetian basins	45°59'N	12°54'E
2	Bacchiglione	Bacchiglione	20	A		45°37'N	11°26'E

3	Sarca	Po	8	A		45°96'N	10°92'E
4	Chiese	Po	4	A		45°89'N	10°60'E
5	Adda	Po	12	A		45°40'N	09°49'E
6	Ticino	Po	12	A		45°40'N	08°92'E
7	Toce	Po	15	A		46°07'N	08°26'E
8	Agogna	Po	15	A		45°69'N	08°42'E
9	Sesia	Po	15	A		45°57'N	08°18'E
10	Orco	Po	15	A		45°33'N	07°72'E
11	Dora Riparia	Po	15	A	Po basin	45°08'N	07°40'E
12	Lemina	Po	11	A		44°91'N	07°31'E
13	Pellice	Po	13	A		44°78'N	07°51'E
14	Maira	Po	15	A		44°46'N	07°37'E
15	Vernetto	Po	15	A		44°99'N	08°02'E
16	S. Bernardo	Po	12	A		44°38'N	07°91'E
17	Bormida	Po	15	A		44°39'N	08°21'E
18	Orba	Po	15	A		44°62'N	08°60'E
19	Scrivia	Po	15	A		44°80'N	08°97'E

20	Tesino	Tesino	10	A	Middle Adriatic	42°57'N	13°38'E
21	Burano	Burano	10	A		43°34'N	12°40'E

22	Peillon	Peillon	15	SF	France	43°46'N	07°22'E

23	Barbaira	Nervia	15	L	West Liguria	43°53'N	07°38'E
24	Oxentina	Argentina	15	L		43°91'N	07°85'E
25	Arroscia	Centa	15	L		44°03'N	07°58'E
26	Barelli	Sciusa	15	L		44°23'N	08°36'E

27	Trexenda	Quiliano	15	L	Central Liguria	44°17'N	08°25'E
28	Lerone	Lerone	15	L		44°23'N	08°39'E
29	Sardorella	Polcevera	15	L		44°29'N	08°54'E

30	Gentile	Gentile	15	L	East Liguria	44°35'N	09°16'E
31	Sturla	Entella	15	L		44°23'N	09°22'E
32	Di Piazza	Deiva	15	L		44°22'N	09°52'E
33	Graveglia	Magra	15	L		44°09'N	09°46'E

34	Merse	Ombrone	10	T	Tuscano-Latium	43°14'N	11°18'E
35	Farma	Ombrone	11	T		43°07'N	11°26'E
36	Zancona	Ombrone	15	T		42°89'N	11°54'E
37	Ente	Ombrone	10	T		42°87'N	11°54'E
38	Albegna	Albegna	4	T		42°79'N	11°51'E
39	Tevere	Tevere	18	T		43°33'N	12°07'E

The population number, river, river basin, number of individuals analyzed, the drainage system ("Drainage"), and the GPS coordinates of the sampling sites are given for each population. A = Adriatic basins; SF = South France; L = Ligurian basins; T = Tuscany basins.

### Molecular analyses

Total DNA was extracted using a standard extraction kit (DNeasy tissue kit, Qiagen, Valencia, CA, USA). All individuals were genotyped at eight highly informative microsatellite loci isolated for *T. souffia *(*Lsou05*, *Lsou08*, *Lsou09*, *Lsou10*, *Lsou11*, *Lsou19*, *Lsou21*, *Lsou34*; [25]). Microsatellites were amplified with fluorescently labeled forward primers (FAM, HEX and NED). Three different sets of markers were used such that all fragments could be separated within a single capillary without overlap and scored unambiguously. The first set included *Lsou05*, *Lsou08 *and *Lsou19*, the second set included *Lsou10 *and *Lsou34*, and the third set included *Lsou09*, *Lsou11 *and *Lsou21*. Polymerase chain reaction was carried out in 10 μL reaction volumes containing 5 μL QIAGEN Multiplex PCR Master mix, 3 μL ddH_2_O, 1 μL DNA (20 ng/μL) and 1 μL primer mix (2 pmol/μL per primer) on a Veriti thermal cycler (Applied Biosystems). The PCR profile started with an initial denaturation step at 95°C for 15 min, followed by 30 cycles of 30 s at 94°C, 90 s at 56°C, 90 s at 72°C and ended with a final extension of 10 min at 72°C. Fragment lengths were determined with the internal size marker Genescan-500 ROX (Applied Biosystems) on an AB 3130 automated capillary sequencer (Applied Biosystems) and scored with GeneMapper (Applied Biosystems).

### Statistical analyses

Allele binning was performed with TANDEM[26]. The software MSA[27] was used to determine mean microsatellite allele numbers (A_N_), observed heterozygosities (H_o_) and expected heterozygosities (H_e_) within populations. GENEPOP version 3.2a [28] was employed to estimate deviations from Hardy-Weinberg Equilibrium (HWE) across populations (within loci) and across loci (within populations) using the probability test, with 10000 dememorization steps, 100 batches and 5000 iterations per batch based on the approach by Guo and Thompso [29]. Computation of pair wise multilocus F_ST _values [30] among populations, as well as R_ST _and F_IS _values was performed using the software ARLEQUIN version 3.1 [31] with 1000 permutation and an allowing a level of missing data of 0.05. Allele frequencies, a pair wise population matrix of Nei's genetic distance were computed by GENEALEX[32].

A neighbor joining (NJ; [33]) population tree was generated based on Cavalli-Sforza & Edwards chord distances (*D*_C_) [34], which were calculated in PHYLIP version 3.67 [35]. Following the recommendation of Van Dongen [36] with respect to relatively low numbers of loci, 10000 bootstrap pseudo-replicates were performed over individuals rather than loci. Bootstrap replicates were generated using the application SEQBOOT (available in PHYLIP package).

Factorial Correspondence Analysis (FCA, [37]), which displays the genetic similarity among samples in a three-dimensional graphical space, and an assessment of the genetic variability in each population were undertaken using the software GENETIX version 4.02 [38]. STRUCTURE version 2.2 [39] was used to determine the population structure by Bayesian clustering analysis, with a burn-in of 5 × 10^4 ^and 10^6 ^iterations, and not using prior population information. The most likely value for the number of populations (*K*) was determined by the "*L*(*K*)" method suggested by Evanno *et al. *[40], varying *K *from a minimum of one to a maximum of 11 and always performing 10 runs for each value of *K*.

Hierarchical analysis of molecular variance (AMOVA), based on allele frequency information [41], was carried out using ARLEQUIN. Variance components were extracted for the (*i*) among group, (*ii*) between sampling sites within groups, and (*iii*) within sampling sites hierarchical levels. The populations were partitioned into groups according to their geography and following the outcomes of the pair wise multilocus F_ST _calculations, the FCA, the NJ tree and the Bayesian clustering analysis.

## Results

Two of the microsatellite markers could not be successfully amplified in a small number of populations and were coded as missing data for the subsequent analyses: *Lsou09 *in populations 20 and 21, and *Lsou11 *in populations 20, 21, 22 and 26 (Additional file 1). All eight microsatellite loci were polymorphic, the number of alleles ranged from 9 to 17 at any single locus (mean 13.4). Mean expected heterozygosity per population ranged from 0.12 (s. d. 0.27, pop. 38) to 0.6 (s. d. 0.21, pop. 3), and averaged 0.45 across all loci and populations. In general, the differences between expected and observed heterozygosities were low, ranging from 0 to 0.16 (mean 0.05) (see Additional file 1 for more details).

Significant departures from Hardy-Weinberg equilibrium (HWE) were observed in a number of populations and mainly in two loci, *Lsou09 *and *Lsou11*. This was most likely due to problems with the amplification of these loci in a small subset of samples (see above), which resulted in missing data in the evaluation of the departure from HWE. In the remaining loci the departures from HWE were due to an excess of homozygosity (Additional file 1).

F_ST _within loci and among populations were highest for *Lsou10 *(0.46), *Lsou11 *(0.5) and *Lsou34 *(0.4) (Additional file 2). Allele frequencies of all populations belonging to the Tuscano-Latium district showed a genetic pattern different from the other populations (Additional file 3). Populations from middle Adriatic (20 and 21) had characteristic allelic patterns in *Lsou08 *and *Lsou21*, while population 22, constituted by *T. souffia*, was characterized by a private allelic pattern in loci *Lsou05*, *Lsou09*, *Lsou19 *and *Lsou34*.

Nei's distances among populations also indicate the great difference between the single population of *T. souffia souffia *and the populations of *T. muticellus*. Six principal groups of populations were recovered by F_ST _and R_ST _(Additional file 2) and Nei's distance (not shown): (*i*) the largest group included the specimens from the Venetian basins (pops. 1 and 2), the Po basin (pops. 3 to 19) plus the populations of Central Liguria (pops. 27, 28 and 29); (*ii*) a second group included two populations from West Liguria (pops. 23 and 24), which showed the lowest Nei's distance to populations from the opposite side of Apennine chain (pops. 15, 16 and 17) belonging to the Padano-Venetian district; (*iii*) the third group included specimens from middle Adriatic and showed large distances to all the others populations; (*iv*) individuals from Tuscano-Latium district form the fourth group (pops. 34 to 39); (*v*) individuals from East Liguria (pops. 30 to 33), which showed the smallest distance to populations of Tuscany, formed another group. The remaining two populations from Western Liguria (pops. 25 and 26) showed F_ST _and Nei's distance values intermediate between the Padano-Venetian populations and the other two Western Ligurian populations (pops. 23 and 24); (*vi*) the last group is equivalent to population 22 (*T. souffia souffia*) and was excluded from the further analyses - also, because there was no sign of introgression with neighboring *T. muticellus *populations in a STRUCTURE analysis.

**Figure 2:**
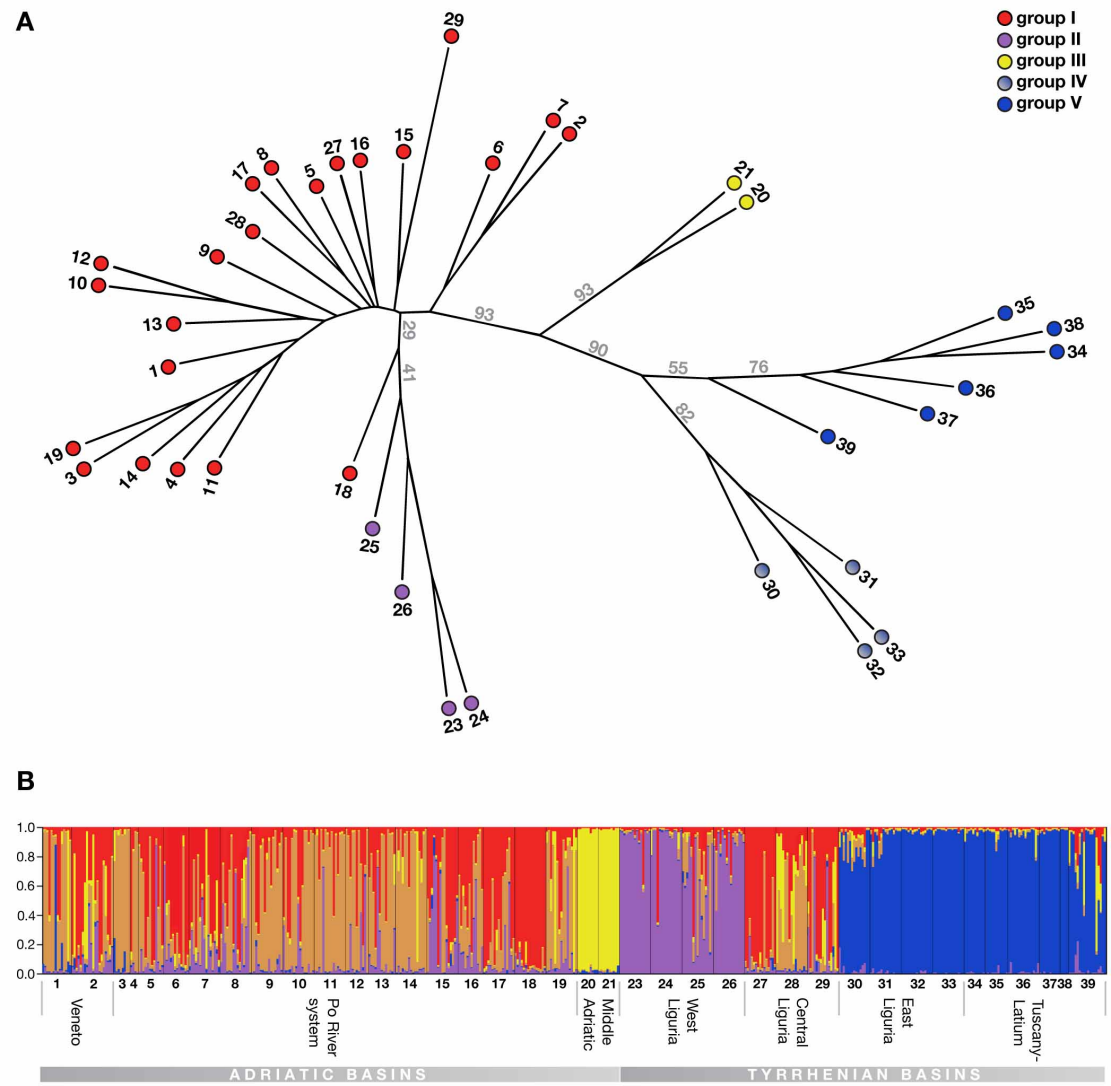
**Population structure in the Italian vairone**. (*a*) Unrooted neighbor joining population tree based on Cavalli-Sforza & Edwards chord distances (*D*_C_) calculated with PHYLIP. The populations from Veneto (pops. 1 and 2), the Po River system (pops. 3 to 19) and Central Liguria (pops. 27 to 29) form a clade that also includes the populations from West Liguria (pops. 23 to 26); the two populations from the Middle Adriatic basins (pops. 20 and 21) form a well supported monophyletic group, just as the populations from East Liguria (pops. 30 to 33) and from Tuscany (pops. 34 to 39). Numbers above the branches correspond to bootstrap values (10000 pseudo-replicates over individuals). (*b*) Results from the population assignment test with STRUCTURE (*K *= 5). Again, the individuals from Veneto, the Po River system and Central Liguria are clustered together, their genomes being admixed of two distinct genotype classes. Individuals from the Middle Adriatic basins and from West Liguria fall into two respective clusters; individuals from East Liguria and from Tuscany form a single cluster.

Because of the unsuccessful amplification of two loci in a few populations, two NJ trees were constructed, one with all populations without the two not amplified loci (Fig. 2a), and one with all loci without the populations with missing amplifications (not shown). The general topology of the two trees was in agreement and in congruence with the pattern of the observed F_ST _values among populations. Four main groups were recovered: (*i*) the populations of the Padano-Venetian district (pops. 1 to 19) plus Central Liguria (pops. 27 to 29) and including a clade formed by the populations from Western Liguria (pops. 23 to 26); (*ii*) the two populations from the Middle Adriatic (pops. 20 and 21); (*iii*) populations from East Liguria (pops. 30 to 33); and (*iv*) populations from the Tuscano-Latium district (pops. 34 to 39). These clades were supported by high bootstrap values (Fig. 2a).

In the FCA plot of the entire data set, three main clusters of individuals were resolved: (*i*) the Tuscano-Latium and East Ligurian populations; (*ii*) the populations from the Middle Adriatic; and (*iii*) populations belonging to the Po basin, the Venetian basins, and the West and Central Ligurian basins (Additional file 4a). The first three axes explained 11.74% of the total genetic variance. We then divided the data set according to the two sea catchments. The FCA conducted on the Adriatic basins showed the distinction between the middle Adriatic basin and the rest of the Padano-Venetian district (Additional file 4b). The FCA conducted on the Tyrrhenian basins showed a structure that follows the grouping explained by both the NJ tree and the pair wise multilocus F_ST _values, with a group representing the West Ligurian populations, a group representing the Central Ligurian populations, a group formed by the East Ligurian populations, and another one by the Tuscano-Latium populations (Additional file 4c).

The most probable number of *K *populations in the Bayesian clustering analysis was 5 when applying the "*L(K)*" method suggested by Evanno *et al. *[40] (Fig. 2b; Additional file 5). With *K *= 5, a population structure with four principal groups was recovered: (*i*) individuals from the Veneto and Po River populations (pops. 1 to 19) and Central Liguria (pops. 27 to 29); (*ii*) individuals from the Middle Adriatic populations (pops. 20 and 21); (*iii*) individuals from West Liguria (pops. 23 to 26); and (*iv*) specimens from East Liguria (pops. 30 to 33) and Tuscany-Latium (pops. 34 to 39). The genomes of the first group consisted of two distinct genetic pools.

In order to quantify population genetic structuring within and among populations a hierarchical AMOVA was computed grouping the populations into five groups following the grouping suggested by F_ST _analysis, the FCA, the NJ tree and the Bayesian assignment tests. Twenty-nine percent of the variation occurred among groups, 7.7% among populations within groups, and 62.8% of the variation was attributed within populations (Additional file 6). F_IS _values are shown in Additional file 7.

## Discussion

### Population structure and phylogeography of the Italian vairone

Our phylogeographic and population genetic analyses based on eight microsatellite markers identified five principal clusters of populations in the Italian vairone. We used information on the geographic origin of the populations, the population NJ tree, the FCA plots as well as the results from the Bayesian assignment test to infer these groups. We note, however, that - despite of the overall support for this grouping - certain analyses resulted in a somewhat deviating picture. These cases are discussed below.

The most widespread group I corresponds to the Padano-Venetian district and includes populations from two Upper Adriatic basins (pops. 1 and 2) and the Po drainage (pops. 3 to 19). The geographic extension of these populations reflects the expansion of the Po river basin up to the Meso-Adriatic depression, which occurred during the last glacial maximum coinciding with the eustatic sea level regression by 120 m [42,43]. Interestingly, three populations of Central Ligurian basins are also included in this group (pops. 27 to 29), although these rivers drain into the upper Tyrrhenian Sea. This clustering of Central Ligurian populations with those from the Po system in all analyses clearly suggests a limited period of "permeability" of the Apennine barrier, allowing gene flow between the Po drainage and Central Liguria. Group I was consistently recovered in the STRUCTURE analyses and in the FCA plots. Yet, in the population tree, it did not come out as monophyletic, since another group (group II) was resolved within group I (see below). The population assignment tests with STRUCTURE further indicated that the genomes of group I individuals are composed of two genetic pools. This may be explained by the existence of at least two isolated lineages prior to the last glacial maximum that became admixed secondarily.

Group II includes four populations from West Liguria (pops. 23 to 26) and shows affinities with group I, *i.e. *the fishes from the Padano-Venetian district. In the population tree, group II was actually resolved within group I populations, while in the Bayesian population assignment tests, group II formed a unique cluster (Fig. 2). It is evident, though, that some individuals show the genotypic signature of group I. This pattern could be the result of introgression of Padano-Venetian genomes from the Central Ligurian populations into an older, isolated western Ligurian stock. Alternatively, the West Ligurian basins might have been colonized directly from the upper reaches of the Po system via river capture, as is indicated by the close genetic affinity of population 18 (river Orba) from the Po system to the geographically close populations of West Liguria (Figs. 1, 2). The individual profile of the West Ligurian populations in the STRUCTURE analyses might then be the result of repeated bottlenecks as a consequence of westward dispersal.

Group III consists of two populations only (pops. 20 and 21) that were sampled at the southern edge of the distribution range of *T. muticellus *in two rivers draining into the Adriatic Sea (Tesino and Burano). These two populations clustered together in all analyses and appeared equidistant from groups I and II, group VI and group V, respectively, in the population tree and the FCA plot (Fig. 2; Additional file 4). The Padano-Venetian ichthyogeographic district, which also comprises the Middle Adriatic basin including rivers Tesino and Burano, has been defined based on the extension of the Po basin during the last glacial maximum due to the lowering of the sea level [12] (Fig. 1c). Now, our new analyses of the Italian vairone contradict the view of a close affinity of the Middle Adriatic populations to the Padano-Venetian ones, but support the existence of an isolated Middle Adriatic stock, instead.

Group IV from East Liguria (pops. 30 to 33) was resolved as separate cluster in the population tree and in the FCA plot. Yet, the Bayesian analyses of population structure suggest its close affinity to populations from the Tuscano-Latium district (group V). An origin of the East Ligurian *T. muticellus *in Tuscany has already been suggested based on mtDNA markers [17].

Group V corresponds to the populations of the Tuscano-Latium district (pops. 34 to 39) that were sampled in the Apennine in the upper reaches of the Ombrone, Albegna and Tevere rivers, which all drain into the middle Tyrrhenian Sea. This group was recovered in the FCA plots and the population NJ tree. In the STRUCTURE analyses, it clusters with group IV individuals. The high levels of genetic similarity between the six Tuscano-Latium populations suggest that the three basins sampled shared contact events. These are likely to have occurred in the upper or middle watercourses, where hydrogeographic structures appear conducive for historical transitory river captures. Because of the large distance between the estuaries of the Ombrone and Albegna rivers to the Tevere river and the bathymetric profile of the Tyrrhenian Sea, a connection via downstream river confluence, *e.g. *during the last glacial maximum when sea levels were substantially lower than at present, appears highly unlikely.

Previous mitochondrial DNA based studies of the Italian vairone [17,44] identified three distinct clusters, which are, overall, in agreement to the ones identified here. Using a smaller set of samples, Zaccara *et al. *[17] found a clear separation between *T. muticellus *populations from Tuscany (group V in our study), East Liguria (group IV) and the Padano-Venetian district (group I). Interestingly, their specimens from Western Liguria (group II) clustered together with populations from the Po drainage, just as they do in our population tree (Fig. 2a). However, neither were Central Ligurian populations included in the study of Zaccara *et al. *[17] (these belong, according to our new analyses, to group I), nor were the Middle Adriatic populations (our group III) studied. Thus, with 509 specimens from 38 populations covering the species' entire distribution range, the present work is the most extensive phylogeographic study of *T. muticellus*.

### A biogeographic scenario for Italian freshwater fishes

The vairone has previously been studied in the context of postglacial re-colonization scenarios in and around the Alps (see *e.g.*, [14,15,17]). There are several reasons that make this species suitable for phylogeographic research. First, the vairone occurs in all major river systems draining off the Alps (Danube, Rhine, Rhône, Po) as well as in many small rivers draining directly into the Mediterranean Sea. Second, the vairone is restricted to certain habitat types within these rivers, making recent large-scale migrations highly unlikely [45]. Third, in most parts of its distribution range, there is no evidence for human-induced faunal translocation, so that the species' present population structure should display its phylogeographic history. In the present study, we use the Italian vairone (*T. muticellus*) as a model to infer the phylogeography of Italian freshwater fish species, suggesting a scenario that might serve as a model for other species as well. Specifically, we were interested in the ways by which the vairone, a reophilic species, was able to colonize different river basins through different routes. This should allow us to suppose recent changes in the shape and connection of the hydrographic basins at regional and local scale.

A first outcome from our study is that the populations of *T. muticellus *are highly structured and fall into five distinct groups, which correspond to geographic areas and drainage systems (see above). Overall, our analyses highlight the existence of strong barriers between geographic areas and major drainage systems, which is reflected in the discrete clustering of populations according to drainage systems, as well as in the significant F_ST_-values between populations even within drainages (Additional file 2).

In our analyses based on nuclear markers, the Apennine emerges as a strong barrier between populations from PV (pops. 1-19) and TL (pops. 34-39). This is in contrast to previous mtDNA based results. Using the very same samples as we do here, Zaccara *et al. *[17] found mtDNA haplotypes of Tuscano-Latium origin in the Po basin, pointing to a past connection between these two major ichthyogeographic districts. This discrepancy between nuclear and mitochondrial DNA is best explained by a scenario in which some individuals crossed the watershed from the TL district into tributaries of the Po basin. While the descendant lines kept their TL identity in the mtDNA (due to the clonal mode of inheritance of mtDNA), their original nuclear DNA signature disappeared (due to repeated and unidirectional crosses with PV fish leading to a homogenization of the genome towards the native type). This scenario dates the connection between the Padano-Venetian and the Tuscano-Latium ichthyogeographic district in an epoch ancient enough to maintain the genetic differences in the mtDNA, but not in the microsatellites (dated in 0.5 millions years ago by Stefani *et al. *[44]). This outcome also illustrates the importance of studying both mtDNA and ncDNA to infer a species' phylogeographic history.

While, with the eight microsatellite markers applied, we could not detect the obviously more ancient introgression event(s) in *T. muticellus *between the two ichthyogeographic districts, several more recent contacts between vairone populations within the two districts became evident. In the PV district, the contacts between isolated basins, *e.g. *between the Po drainage (pops. 3-19), the Bacchiglione (pop. 2), and the Tagliamento (pop. 1), were possible during periods of glaciation, when the sea level was markedly lowered (see also Salzburger *et al. *[15]). During these periods, the estuary of the Po reached the middle Adriatic pit, connecting the rivers of the entire PV ichthyogeographic district via downstream river confluence (Fig. 1c). The bathymetric profile of the Adriatic Sea, which is a shallow sea with a gently declining profile, made river confluence possible. The Tyrrhenian Sea, on the other hand, is rather deep and has a much steeper profile, so that downstream river confluence is a rather unlikely cause for the observed gene flow between rivers of the TL district. There, historical connections in the middle and upper courses of the rivers, caused by local plate-tectonic events along the Apennine ridge, are most probably responsible for faunal exchange [46,47]. The genesis and the evolution of the Apennine chain favored such kind of exchange, with a wrinkle structure that caused the formation of valleys parallel to the direction of the mountain chain. On the Tyrrhenian side, rivers follow these valleys for long traits, assuming a trend with a North-South orientation, while, on the Adriatic side, rivers are principally orthogonal to the mountain chain. The closing of these valleys could cause the overflowing with the outlet of a river into those of the adjacent valley [48]. Connections between rivers belonging to different sides of a watershed were also suggested for rivers with terminally aligned traces, such as river Lerone and Orba (see Table 1 and Fig. 1) [48]. Such temporal connections between rivers in the past can also explain the phylogeographic pattern observed in the Ligurian basins, where "passover events" occurred from the PV district along the west side of the Alpine chain (pops. 23-26), and from the TL district along the upper Tyrrhenian slope of the Apennine chain (pops. 30-33). The origin of the Middle Adriatic populations (pops. 20 and 21) remains somewhat elusive.

From our analyses at least four different refugia can be delineated, where Italian vairone populations outlived the last glacial maximum: one for the Upper Adriatic basins, one for the Middle Adriatic rivers, one in the Tuscano-Latium area and at least one in (East) Liguria. The areal expansion in the Italian vairone has already been implicated with population bottlenecks (in the refugia) followed by demographic expansions, as a consequence of environmental alterations during these periods [17]. Our new analyses corroborate this view.

The population assignment tests with STRUCTURE indicate the existence of two distinct refugia for the Upper Adriatic basins, since the genomes of the sampled individuals appear admixed from more than one genetic pool. The formation of different genetic pools can be attributed to the cyclic variation of the sea level of the Adriatic Sea during glacial cycles. In periods of maximal glaciation, the Po connected all river systems as far as to the Meso-Adriatic depression (see Fig. 1c); during these cold periods, the typical vairone habitat shifted towards lower altitudes, whereas - during interglacial periods - the present situation with many isolated populations was restored [45]. Several factors may account for the generally high degree of isolation between Italian vairone populations, particularly in the Adriatic drainages. First, suboptimal environmental conditions during cold periods could - although allowing for survival of vairone populations - have inhibited long distance dispersal, thereby limiting gene flow. Such a situation has been reported for the minnow (*Phoxinus phoxinus*), another cold-adapted rheophilic freshwater fish species [49]. Second, the sandy substrate on the bottom of the Adriatic Sea, which is where the rivers drained to during glaciation (see Fig. 1c), appears unsuitable for egg deposition by the vairone. Since the vairone requires gravely, shallow and well-oxygenated waters, proper spawning grounds may only have been available in the middle parts of rivers, again limiting dispersal. Finally, the (temporarily) increased salinity levels close to the river mouths could have led to barriers for downward migration for stenohaline species such as the vairone.

## Conclusion

Taken together, the following phylogeographic scenario arises for *T. muticellus*: an initial *T. muticellus *stock became isolated from its 'Alpine' allies, possibly triggered by the Mediterranean Salinity Crisis some five million years ago [15]. The Italian vairone survived the various glaciation cycles in several refugia, most likely situated in the Upper Adriatic (groups I and II), the Middle Adriatic (group III), (East) Liguria (group IV) and Tuscano-Latium (group V); the latter two show closer affinities (see Fig. 2) and might originate from a common Tyrrhenian stock. This grouping is, overall, in agreement with the two main ichthyogeographic districts, Padano-Venetian and Tuscano-Latium, except for the two populations in the Middle Adriatic, which we identify as additional major district. Although in principle dividing the PV and TL districts, the Apennine barrier was "permeable" at some point, leading to the introgression of mitochondrial haplotypes from Tuscano-Latium into the Po drainage [17]; this can be the result of local events of river capture, as suggested by Ghelardoni [48]. Owing to a more complex phylogeographic history, the *T. muticellus *stocks in the isolated smaller rivers in Liguria do not form a unique cluster. Populations from East Liguria group with those of the Tuscano-Latium district, whereas stocks from Central and West Liguria show affinities to the Padano-Venetian fishes (note that it is unlikely that this situation is due to recent human-induced faunal introductions in Liguria as suggested by Balma *et al. *[50], since most of the Ligurian populations do show private genetic signatures). Future studies in other freshwater taxa will now be necessary to evaluate the universal validity of the "vairone scenario" proposed here.

## Authors' contributions

FM, SZ and WS designed the study; FM and FMM carried out the molecular work; FM, FMM and WS performed the analyses. All authors contributed to the preparation of the manuscript. They read and approved the final version.

## Supplementary Material

Additional file 1 **Number of individuals (N), number of alleles (Na), observed heterozygosity (Ho), expected heterzygosity (He) per population per locus and mean values, FST values per locus and mean values of Na, Ho and He across all loci and all populations, excluding pop. 22 (*T. souffia*)**. Click here for file

Additional file 2 Estimate of FST among population (above diagonal) and Nei's distance (below diagonal). Click here for file

Additional file 3 Allele frequencies. Click here for file

Additional file 4 Supplemental Figure. Click here for file

Additional file 5 Supplemental Figure. Click here for file

Additional file 6 AMOVA results. Click here for file

Additional file 7 FIS values. Click here for file
