# Supplementary material for: Phylogeography of the Italian vairone (Telestes muticellus, Bonaparte 1837) inferred by microsatellite markers: evolutionary history of a freshwater fish species with a restricted and fragmented distribution
Source: BMC Evol Biol. 2010 Apr 27;10:111. doi: 10.1186/1471-2148-10-111 (PMC2868840; doi:10.1186/1471-2148-10-111)
Supplement: Additional file 1 — Number of individuals (N), number of alleles (Na), observed heterozygosity (Ho), expected heterzygosity (He) per population per locus and mean values, FST values per locus and mean values of Na, Ho and He across all loci and all populations, excluding pop. 22 (T. souffia). [file 1471-2148-10-111-S1.PDF]

Additional file 1: Number of individuals (N), number of alleles (Na), observed heterozygosity (Ho), expected heterozygosity (He) per population per locus and mean values, F_{ST} values per locus and mean values of Na, Ho and He across all loci and all populations, excluding pop. 22 (*T. souffia*).

Pop		Lsou05	Lsou08	Lsou19	Lsou10	Lsou34	Lsou09	Lsou11	Lsou21	mean	st. dev.	He_{mean}-Ho_{mean}
**Pop1**	**N**	14	14	14	14	14	11	11	14			
	**Na**	8	6	2	6	4	6	3	6	5.13	1.96	
	**Ho**	0.86	0.64	0.50	0.79	0.57	0.09**	0.09	0.57**	0.57	0.27	0.04
	**He**	0.76	0.75	0.44	0.73	0.67	0.69	0.17	0.76	0.62	0.21	
**Pop2**	**N**	20	20	20	20	18	20	16	20			
	**Na**	4	5	2	2	4	5	3	8	4.13	1.96	
	**Ho**	0.65	0.55	0.20	0.65	0.78	0.2**	0.12**	0.85	0.61	0.23	-0.10
	**He**	0.59	0.66	0.18	0.47	0.68	0.38	0.32	0.80	0.51	0.21	
**Pop3**	**N**	8	8	8	8	8	8	6	8			
	**Na**	5	6	2	2	4	5	3	4	3.88	1.46	
	**Ho**	0.75	0.88	0.50	0.25	0.88	0.50	0.50	0.50	0.59	0.22	0.00
	**He**	0.75	0.77	0.50	0.22	0.73	0.66	0.57	0.55	0.59	0.18	
**Pop4**	**N**	4	4	4	4	4	3	4	4			
	**Na**	5	3	2	1	2	1	3	4	2.63	1.41	
	**Ho**	1.00	0.25	0.50	0.00	0.25	0.00	0.50	0.50	0.38	0.33	0.00
	**He**	0.69	0.53	0.50	0.00	0.22	0.00	0.41	0.66	0.38	0.27	
**Pop5**	**N**	12	12	12	11	12	5	12	12			
	**Na**	5	3	2	2	4	3	2	6	3.38	1.51	
	**Ho**	0.67	0.17	0.33	0.36	0.5**	0.40	0.08**	0.83	0.46	0.24	0.00
	**He**	0.65	0.16	0.28	0.30	0.70	0.54	0.33	0.72	0.46	0.22	
**Pop6**	**N**	12	12	12	12	12	11	11	12			
	**Na**	3	6	2	3	5	3	3	8	4.13	2.03	
	**Ho**	0.58	0.67	0.42	0.42	0.92	0.18**	0.09	0.92	0.57	0.30	-0.05
	**He**	0.57	0.56	0.33	0.34	0.77	0.52	0.24	0.82	0.52	0.21	
**Pop7**	**N**	15	15	15	15	15	12	12	15			
	**Na**	7	5	2	3	6	4	2	8	4.63	2.26	
	**Ho**	0.67	0.33**	0.13	0.53	0.60	0.25	0.17	0.80	0.45	0.26	0.08
	**He**	0.70	0.59	0.23	0.52	0.66	0.35	0.38	0.82	0.53	0.20	
**Pop8**	**N**	15	15	15	15	15	14	12	15			
	**Na**	4	5	2	2	5	3	2	9	4.00	2.39	
	**Ho**	0.87	0.33**	0.13	0.33	0.67	0.21**	0.08	0.67	0.46	0.32	0.01
	**He**	0.65	0.61	0.12	0.28	0.71	0.40	0.22	0.76	0.47	0.24	
**Pop9**	**N**	15	15	15	15	15	12	13	15			
	**Na**	7	8	2	2	4	5	1	7	4.50	2.67	
	**Ho**	0.80	0.60	0.40	0.27	0.60	0.25**	0.00	0.6**	0.44	0.28	0.02
	**He**	0.75	0.61	0.32	0.23	0.54	0.57	0.00	0.72	0.47	0.26	
**Pop10**	**N**	15	15	15	14	15	11	11	15			
	**Na**	7	10	2	4	4	4	1	6	4.75	2.87	
	**Ho**	0.87	0.67	0.20	0.71	0.67	0.45	0.00	0.87	0.55	0.31	-0.03
	**He**	0.74	0.68	0.18	0.61	0.73	0.44	0.00	0.81	0.53	0.29	
**Pop11**	**N**	15	15	15	15	15	11	12	15			
	**Na**	8	7	2	4	4	4	2	7	4.75	2.31	
	**Ho**	0.87	0.53	0.33	0.33	0.60	0.18**	0.08**	0.53**	0.53	0.22	-0.05

	**He**	0.77	0.67	0.36	0.30	0.64	0.44	0.08	0.65	0.49	0.23	
**Pop12**	**N**	11	11	11	11	11	10	7	11			
	**Na**	7	4	2	4	4	6	2	5	4.25	1.75	
	**Ho**	0.73	0.64	0.36	0.55	0.55	0.5**	0.14	0.82	0.54	0.23	0.05
	**He**	0.78	0.59	0.30	0.62	0.73	0.73	0.34	0.63	0.59	0.18	
**Pop13**	**N**	13	13	13	13	13	10	10	13			
	**Na**	7	4	2	4	5	6	2	9	4.88	2.42	
	**Ho**	0.62	0.31	0.23	0.46	0.69	0.4**	0.00	0.69	0.43	0.26	0.07
	**He**	0.67	0.28	0.39	0.39	0.64	0.70	0.18	0.78	0.50	0.22	
**Pop14**	**N**	15	15	15	14	15	10	12	15			
	**Na**	5	8	2	3	4	3	4	8	4.63	2.26	
	**Ho**	0.67	0.67	0.47	0.36	0.80	0.1**	0.25	0.87	0.58	0.23	-0.02
	**He**	0.69	0.71	0.49	0.48	0.66	0.40	0.23	0.81	0.56	0.19	
**Pop15**	**N**	15	15	15	12	15	11	8	15			
	**Na**	7	9	1	4	4	6	3	8	5.25	2.71	
	**Ho**	0.87	0.80	0.00	0.17**	0.33	0.36	0.13	0.87	0.48	0.36	0.02
	**He**	0.66	0.77	0.00	0.55	0.43	0.56	0.23	0.75	0.49	0.27	
**Pop16**	**N**	12	12	12	12	12	10	6	11			
	**Na**	6	5	2	4	2	6	2	7	4.25	2.05	
	**Ho**	1.00	0.50	0.08**	0.50	0.33	0.4**	0.00	0.64	0.49	0.33	-0.03
	**He**	0.64	0.47	0.08	0.54	0.28	0.72	0.28	0.70	0.46	0.23	
**Pop17**	**N**	15	15	15	15	15	14	11	14			
	**Na**	9	6	2	4	5	6	2	6	5.00	2.33	
	**Ho**	0.80	0.87	0.27	0.33**	0.60	0.36**	0.00	0.64	0.53	0.33	0.04
	**He**	0.77	0.72	0.32	0.50	0.68	0.67	0.17	0.73	0.57	0.22	
**Pop18**	**N**	15	15	15	12	15	14	7	15			
	**Na**	3	7	1	2	3	3	3	7	3.63	2.20	
	**Ho**	0.53	0.67	0.00	0**	0.60	0.21**	0.14**	0.87	0.53	0.32	-0.05
	**He**	0.46	0.77	0.00	0.28	0.44	0.57	0.50	0.84	0.48	0.27	
**Pop19**	**N**	15	15	15	15	15	9	10	15			
	**Na**	6	5	3	2	2	1	3	6	3.50	1.93	
	**Ho**	0.67	0.67	0.40	0.40	0.33	0.00	0**	0.80	0.47	0.27	-0.03
	**He**	0.56	0.68	0.56	0.32	0.36	0.00	0.34	0.65	0.43	0.23	
**Pop20**	**N**	10	10	10	10	10	0	0	10			
	**Na**	4	3	1	2	4	0	0	3	2.13	1.64	
	**Ho**	0.80	0.30	0.00	0.20	0.40	0.00	0.00	0.40	0.26	0.28	0.00
	**He**	0.68	0.27	0.00	0.42	0.35	0.00	0.00	0.41	0.26	0.25	
**Pop21**	**N**	10	10	10	9	9	0	0	10			
	**Na**	4	3	2	3	3	0	0	2	2.13	1.46	
	**Ho**	0.40	0.20	0.20	0.33	0.33	0.00	0.00	0.30	0.22	0.15	0.02
	**He**	0.47	0.19	0.32	0.44	0.29	0.00	0.00	0.26	0.24	0.18	
**Pop22**	**N**	15	15	15	15	15	5	0	15			
	**Na**	2	5	4	1	5	2	0	1	2.50	1.93	
	**Ho**	0.40	0.46**	0.33	0.00	0.73	0.00	0.00	0.00	0.21	0.29	0.13
	**He**	0.39	0.74	0.55	0.00	0.70	0.32	0.00	0.00	0.34	0.31	
**Pop23**	**N**	15	15	15	15	15	5	3	15			
	**Na**	4	1	1	1	1	4	3	4	2.38	1.51	
	**Ho**	0.73	0**	0.00	0.00	0.00	0.40	0.33	0.27**	0.24	0.30	0.04

	**He**	0.68	0.00	0.00	0.00	0.00	0.48	0.50	0.60	0.28	0.31	
**Pop24**	**N**	15	15	15	15	15	13	9	15			
	**Na**	3	2	1	1	3	4	4	4	2.75	1.28	
	**Ho**	0.73	0**	0.00	0.00	0.13	0**	0**	0.53	0.28	0.33	0.03
	**He**	0.57	0.12	0.00	0.00	0.18	0.49	0.62	0.46	0.31	0.26	
**Pop25**	**N**	15	15	15	15	15	12	11	15			
	**Na**	5	5	2	3	3	3	2	5	3.50	1.31	
	**Ho**	0.73	0.60	0.00	0.47	0.47	0.25	0.00	0.73	0.41	0.30	0.05
	**He**	0.62	0.67	0.12	0.37	0.49	0.49	0.17	0.76	0.46	0.23	
**Pop26**	**N**	15	15	15	15	15	9	0	15			
	**Na**	4	3	2	2	2	3	0	4	2.50	1.31	
	**Ho**	0.60	0.73	0.20	0.53	0.33	0.78	0.00	0.53	0.46	0.27	-0.05
	**He**	0.54	0.65	0.18	0.39	0.28	0.65	0.00	0.63	0.42	0.25	
**Pop27**	**N**	15	15	15	15	15	15	6	15			
	**Na**	7	8	2	4	4	4	4	5	4.75	1.91	
	**Ho**	0.73	0.80	0.13	0.53	0.73	0.53	0.17**	0.80	0.61	0.24	-0.01
	**He**	0.79	0.78	0.12	0.59	0.59	0.55	0.60	0.74	0.60	0.21	
**Pop28**	**N**	15	15	15	15	15	15	10	15			
	**Na**	5	5	1	2	3	3	4	4	3.38	1.41	
	**Ho**	0.53	0.80	0.00	0.13	0.73	0.20	0.30	0.53	0.40	0.29	-0.01
	**He**	0.48	0.73	0.00	0.12	0.58	0.18	0.42	0.67	0.40	0.27	
**Pop29**	**N**	15	15	15	15	15	12	14	15			
	**Na**	4	8	1	4	3	6	6	5	4.63	2.13	
	**Ho**	0.60	0.87	0.00	0.40	0.60	0.17**	0.29**	0.27**	0.49	0.32	0.02
	**He**	0.56	0.77	0.00	0.35	0.58	0.69	0.64	0.52	0.51	0.24	
**Pop30**	**N**	15	15	15	15	15	13	11	15			
	**Na**	4	5	3	5	2	9	4	3	4.38	2.13	
	**Ho**	0.53	0.4**	0.53	0.53	0.20	0.38**	0.09**	0.07**	0.45	0.17	0.02
	**He**	0.51	0.64	0.56	0.56	0.18	0.73	0.43	0.13	0.47	0.21	
**Pop31**	**N**	15	15	15	15	15	12	13	15			
	**Na**	4	7	2	3	1	9	6	2	4.25	2.82	
	**Ho**	0.80	0.6**	0.33	0.13	0.00	0.75	0.23**	0.07**	0.40	0.36	0.05
	**He**	0.59	0.78	0.46	0.24	0.00	0.81	0.70	0.06	0.46	0.32	
**Pop32**	**N**	15	15	15	15	15	11	8	15			
	**Na**	3	4	2	2	2	7	8	1	3.63	2.56	
	**Ho**	0.40	0.60	0.20	0**	0.20	0.64**	0.5**	0.00	0.28	0.23	0.17
	**He**	0.42	0.61	0.36	0.32	0.28	0.80	0.81	0.00	0.45	0.28	
**Pop33**	**N**	15	15	15	15	15	12	8	15			
	**Na**	5	7	3	2	3	7	4	2	4.13	2.03	
	**Ho**	0.80	0.73	0.4**	0.07	0.53	0.75	0**	0.20	0.51	0.31	0.01
	**He**	0.59	0.82	0.52	0.18	0.50	0.72	0.66	0.18	0.52	0.23	
**Pop34**	**N**	10	10	10	10	10	7	5	10			
	**Na**	2	3	2	2	2	8	6	4	3.63	2.26	
	**Ho**	0.20	0.10	0.1**	0**	0.20	0.43**	0.6**	0.40	0.23	0.13	0.18
	**He**	0.18	0.27	0.10	0.32	0.18	0.83	0.80	0.61	0.41	0.29	
**Pop35**	**N**	11	11	11	11	11	8	6	11			
	**Na**	3	4	1	2	1	6	5	2	3.00	1.85	
	**Ho**	0.45	0.18	0.00	0.00	0.00	0.50	0.33**	0.36	0.21	0.22	0.11

	He	0.43	0.32	0.00	0.17	0.00	0.58	0.74	0.40	0.33	0.26	
**Pop36**	N	15	15	15	15	15	14	12	15			
	Na	4	3	1	2	1	10	9	3	4.13	3.48	
	Ho	0.53	0.60	0.00	0.07**	0.00	0.71**	0.5**	0.53	0.33	0.31	0.11
	He	0.52	0.58	0.00	0.36	0.00	0.80	0.75	0.55	0.44	0.31	
**Pop37**	N	10	10	10	10	10	7	6	10			
	Na	4	3	1	2	1	6	2	2	2.63	1.69	
	Ho	0.80	0.40	0.00	0**	0.00	0.71	0.00	0.50	0.34	0.35	0.01
	He	0.59	0.34	0.00	0.42	0.00	0.74	0.28	0.50	0.36	0.26	
**Pop38**	N	4	4	4	4	4	3	3	4			
	Na	2	1	1	1	1	5	1	1	1.63	1.41	
	Ho	0.25	0.00	0.00	0.00	0.00	1.00	0.00	0.00	0.16	0.35	-0.03
	He	0.22	0.00	0.00	0.00	0.00	0.78	0.00	0.00	0.12	0.27	
**Pop39**	N	18	18	18	18	18	4	6	18			
	Na	6	4	2	3	4	5	2	4	3.75	1.39	
	Ho	0.67	0.44	0.06**	0.50	0.61	0.75	0.33	0.72	0.58	0.15	-0.08
	He	0.68	0.38	0.05	0.59	0.64	0.78	0.28	0.58	0.50	0.24	
	F_{ST}	0.260	0.313	0.355	0.455	0.404	0.286	0.496	0.320	0.361		

**mean Na**	3.80	SD	0.94
**mean Ho**	0.44	SD	0.13
**mean He**	0.45	SD	0.11

** loci with a significant departure from the Hardy-Weinberg equilibrium.
