# Supplementary material for: Phylogeography of the Italian vairone (Telestes muticellus, Bonaparte 1837) inferred by microsatellite markers: evolutionary history of a freshwater fish species with a restricted and fragmented distribution
Source: BMC Evol Biol. 2010 Apr 27;10:111. doi: 10.1186/1471-2148-10-111 (PMC2868840; doi:10.1186/1471-2148-10-111)
Supplement: Additional file 2 — Estimate of FST among population (above diagonal) and Nei's distance (below diagonal). [file 1471-2148-10-111-S2.PDF]

Additional file 2: Estimate of F_{ST} among population (above diagonal) and Nei's distance (below diagonal).

	Pop01	Pop02	Pop03	Pop04	Pop05	Pop06	Pop07	Pop08	Pop09	Pop10	Pop11	Pop12	Pop13	Pop14	Pop15	Pop16	Pop17	Pop18	Pop19
Pop1	-	0.06	0.08	0.08	0.09	0.08	0.05	0.09	0.06	**0.02**	0.04	0.04	0.07	0.06	0.11	0.11	0.05	0.14	0.13
Pop2	0.12	-	0.20	0.15	0.09	0.05	**0.02**	0.07	0.08	0.04	0.09	0.09	0.09	0.12	0.08	0.11	0.08	0.12	0.16
Pop3	0.25	0.37	-	**0.10**	0.17	0.14	0.16	0.15	0.11	0.13	0.08	0.13	0.14	0.09	0.22	0.21	0.10	0.26	0.13
Pop4	0.21	0.21	0.23	-	0.14	0.12	0.15	**0.07**	0.11	0.09	**0.01**	0.11	**0.01**	0.07	0.14	0.08	0.08	0.16	0.08
Pop5	0.17	0.13	0.29	0.19	-	0.06	0.04	0.05	0.02	0.03	0.06	0.04	**0.03**	0.09	0.15	0.10	0.06	0.18	0.15
Pop6	0.17	0.10	0.30	0.21	0.11	-	0.04	0.05	0.06	0.05	0.07	0.07	0.05	0.09	0.08	0.10	0.05	0.11	0.13
Pop7	0.13	0.05	0.34	0.24	0.10	0.09	-	0.08	0.04	**0.02**	0.08	0.05	0.07	0.12	0.10	0.12	0.05	0.13	0.18
Pop8	0.16	0.09	0.27	0.13	0.09	0.09	0.12	-	0.04	0.03	**0.01**	0.04	0.04	0.06	0.07	0.03	**0.01**	0.11	0.10
Pop9	0.10	0.11	0.23	0.18	0.07	0.09	0.09	0.07	-	0.04	**0.02**	0.03	0.05	0.08	0.14	0.10	**0.02**	0.18	0.13
Pop10	0.08	0.08	0.30	0.18	0.09	0.10	0.08	0.06	0.07	-	0.03	**0.01**	0.03	0.05	0.05	0.06	0.03	0.10	0.13
Pop11	0.10	0.13	0.19	0.08	0.10	0.12	0.14	0.04	0.05	0.06	-	**0.03**	**0.01**	0.05	0.10	0.04	**0.02**	0.13	0.10
Pop12	0.15	0.19	0.34	0.26	0.12	0.17	0.17	0.12	0.10	0.11	0.11	-	0.04	0.05	0.12	0.09	0.03	0.17	0.14
Pop13	0.13	0.14	0.27	0.11	0.08	0.10	0.14	0.08	0.08	0.07	0.05	0.11	-	0.07	0.10	0.05	0.06	0.13	0.13
Pop14	0.16	0.19	0.23	0.15	0.16	0.16	0.23	0.10	0.13	0.11	0.08	0.18	0.13	-	0.13	0.12	0.04	0.17	0.06
Pop15	0.20	0.11	0.39	0.19	0.18	0.12	0.16	0.09	0.17	0.09	0.13	0.22	0.13	0.20	-	0.06	0.08	0.06	0.14
Pop16	0.17	0.16	0.36	0.16	0.13	0.16	0.20	0.08	0.13	0.11	0.08	0.15	0.08	0.19	0.10	-	0.05	0.12	0.11
Pop17	0.18	0.20	0.31	0.26	0.15	0.15	0.21	0.09	0.10	0.13	0.10	0.15	0.14	0.18	0.17	0.12	-	0.11	0.07
Pop18	0.29	0.19	0.48	0.24	0.23	0.18	0.23	0.15	0.25	0.18	0.19	0.32	0.19	0.30	0.11	0.16	0.19	-	0.18
Pop19	0.23	0.20	0.25	0.12	0.21	0.19	0.24	0.13	0.18	0.19	0.13	0.28	0.20	0.10	0.18	0.17	0.21	0.25	-
Pop20	0.80	0.60	0.84	1.10	0.68	0.64	0.66	0.70	0.62	0.79	0.77	0.76	0.83	0.87	0.73	0.76	0.68	0.71	0.82
Pop21	0.87	0.66	0.82	1.10	0.73	0.67	0.74	0.75	0.65	0.87	0.81	0.82	0.89	0.83	0.81	0.81	0.70	0.83	0.77
Pop22	2.38	1.94	2.41	2.45	2.46	2.17	1.80	2.53	2.57	2.30	2.69	3.03	2.56	2.64	1.82	2.61	2.80	2.22	2.37
Pop23	0.39	0.30	0.43	0.22	0.22	0.23	0.26	0.18	0.28	0.26	0.21	0.32	0.18	0.30	0.21	0.15	0.27	0.23	0.24
Pop24	0.49	0.41	0.57	0.39	0.33	0.35	0.38	0.32	0.40	0.40	0.36	0.40	0.27	0.49	0.30	0.25	0.33	0.33	0.46
Pop25	0.19	0.14	0.28	0.22	0.16	0.14	0.16	0.11	0.14	0.13	0.13	0.24	0.17	0.19	0.10	0.12	0.16	0.20	0.14
Pop26	0.56	0.40	0.54	0.40	0.42	0.43	0.43	0.30	0.46	0.47	0.37	0.41	0.40	0.46	0.33	0.26	0.39	0.35	0.31
Pop27	0.23	0.16	0.37	0.27	0.21	0.20	0.22	0.12	0.19	0.15	0.16	0.22	0.20	0.26	0.13	0.14	0.10	0.16	0.24
Pop28	0.24	0.18	0.40	0.17	0.18	0.20	0.19	0.11	0.17	0.12	0.12	0.23	0.16	0.18	0.15	0.20	0.24	0.13	0.21
Pop29	0.29	0.19	0.39	0.32	0.23	0.15	0.22	0.15	0.20	0.19	0.22	0.26	0.26	0.27	0.14	0.21	0.19	0.19	0.22
Pop30	0.63	0.71	0.96	1.16	0.85	0.80	0.70	0.96	0.71	0.75	0.89	0.95	0.94	0.96	0.80	1.05	0.77	0.97	1.04
Pop31	0.64	0.63	1.05	1.21	0.80	0.80	0.62	0.92	0.70	0.72	0.91	0.89	0.95	1.05	0.69	0.97	0.84	0.91	1.07
Pop32	1.15	1.10	1.48	1.69	1.45	1.39	1.09	1.50	1.29	1.40	1.58	1.56	1.69	1.63	1.21	1.59	1.37	1.46	1.41
Pop33	1.20	1.23	1.64	1.97	1.51	1.40	1.16	1.68	1.36	1.47	1.71	1.59	1.74	1.94	1.27	1.64	1.47	1.54	1.81
Pop34	0.96	0.94	1.38	1.50	1.21	1.02	0.98	1.13	1.28	1.10	1.29	1.02	1.26	1.33	1.00	1.16	1.21	1.02	1.40
Pop35	0.85	0.82	1.24	1.16	1.03	0.91	0.79	0.87	1.01	0.92	1.00	0.83	1.10	1.07	0.94	1.00	1.04	0.95	1.12
Pop36	0.78	0.69	1.14	1.15	0.86	0.80	0.66	0.83	0.94	0.82	0.97	0.74	0.91	1.07	0.84	0.89	0.99	0.92	1.16
Pop37	0.92	0.85	1.39	1.39	1.07	0.95	0.81	0.99	1.13	1.02	1.20	0.89	1.08	1.39	1.00	1.06	1.07	0.90	1.55
Pop38	1.17	0.98	1.54	1.80	1.39	1.13	0.99	1.30	1.56	1.28	1.67	1.35	1.55	1.67	1.13	1.50	1.40	1.05	1.68
Pop39	0.88	0.78	1.12	1.22	1.11	0.86	0.78	0.92	1.16	0.93	1.15	1.07	1.07	1.20	0.75	0.94	0.93	0.69	1.11

Values in bold indicate probability values P>0.05

Pop20	Pop21	Pop22	Pop23	Pop24	Pop25	Pop26	Pop27	Pop28	Pop29	Pop30	Pop31	Pop32	Pop33	Pop34	Pop35	Pop36	Pop37	Pop38	Pop39
0.26	0.28	0.42	0.33	0.31	0.12	0.19	0.06	0.16	0.15	0.30	0.31	0.38	0.30	0.34	0.37	0.31	0.29	0.40	0.23
0.25	0.28	0.46	0.32	0.28	0.10	0.17	0.05	0.17	0.13	0.37	0.36	0.44	0.37	0.40	0.44	0.35	0.34	0.45	0.27
0.34	0.35	0.48	0.40	0.40	0.15	0.24	0.14	0.26	0.23	0.40	0.45	0.49	0.40	0.48	0.53	0.46	0.47	0.55	0.35
0.45	0.47	0.55	0.34	0.32	0.15	0.19	0.10	0.16	0.22	0.49	0.54	0.58	0.47	0.58	0.62	0.52	0.52	0.70	0.39
0.34	0.37	0.53	0.30	0.28	0.12	0.21	0.11	0.16	0.18	0.43	0.45	0.53	0.44	0.51	0.53	0.43	0.44	0.57	0.37
0.27	0.29	0.48	0.27	0.27	0.09	0.18	0.08	0.16	0.09	0.39	0.41	0.48	0.39	0.42	0.46	0.38	0.37	0.47	0.28
0.26	0.30	0.45	0.29	0.25	0.11	0.17	0.07	0.16	0.13	0.34	0.34	0.43	0.35	0.40	0.43	0.34	0.33	0.44	0.27
0.31	0.33	0.51	0.24	0.23	0.08	0.12	0.04	0.10	0.10	0.44	0.45	0.51	0.43	0.46	0.47	0.41	0.40	0.53	0.32
0.27	0.29	0.50	0.32	0.30	0.11	0.20	0.08	0.15	0.14	0.39	0.40	0.48	0.40	0.47	0.48	0.42	0.41	0.53	0.35
0.27	0.30	0.45	0.28	0.26	0.08	0.16	0.03	0.09	0.10	0.36	0.36	0.44	0.35	0.39	0.42	0.34	0.33	0.44	0.25
0.30	0.32	0.49	0.25	0.25	0.09	0.14	0.05	0.11	0.14	0.41	0.43	0.49	0.41	0.46	0.47	0.41	0.40	0.52	0.33
0.30	0.32	0.48	0.32	0.31	0.14	0.18	0.06	0.14	0.15	0.39	0.41	0.48	0.38	0.41	0.41	0.34	0.33	0.48	0.30
0.35	0.38	0.51	0.23	0.22	0.12	0.18	0.09	0.13	0.18	0.44	0.46	0.52	0.43	0.48	0.50	0.42	0.42	0.54	0.34
0.29	0.29	0.45	0.31	0.31	0.12	0.16	0.09	0.15	0.16	0.38	0.42	0.45	0.38	0.42	0.44	0.39	0.38	0.48	0.30
0.32	0.36	0.47	0.28	0.26	0.08	0.14	0.04	0.15	0.09	0.41	0.40	0.47	0.38	0.44	0.48	0.40	0.39	0.49	0.25
0.38	0.41	0.55	0.23	0.24	0.07	0.11	0.05	0.17	0.16	0.48	0.50	0.55	0.46	0.52	0.54	0.46	0.46	0.60	0.35
0.26	0.27	0.46	0.24	0.24	0.06	0.11	0.02	0.13	0.09	0.37	0.39	0.44	0.37	0.41	0.42	0.38	0.37	0.47	0.28
0.36	0.41	0.52	0.31	0.29	0.13	0.18	**0.10**	0.10	0.14	0.46	0.46	0.53	0.44	0.47	0.51	0.44	0.41	0.53	0.28
0.33	0.32	0.51	0.29	0.31	0.10	0.12	0.10	0.21	0.15	0.44	0.47	0.50	0.44	0.49	0.53	0.47	0.48	0.56	0.35
-	**0.01**	0.56	0.58	0.56	0.30	0.41	0.27	0.35	0.29	0.44	0.44	0.53	0.44	0.56	0.60	0.52	0.53	0.64	0.40
0.03	-	0.59	0.62	0.60	0.32	0.42	0.29	0.40	0.32	0.48	0.49	0.57	0.48	0.61	0.65	0.57	0.59	0.70	0.43
1.96	2.34	-	0.67	0.65	0.50	0.54	0.43	0.55	0.54	0.39	0.41	0.41	0.31	0.50	0.55	0.47	0.50	0.51	0.42
0.92	1.01	2.71	-	0.06	0.22	0.22	0.26	0.39	0.36	0.64	0.66	0.69	0.62	0.69	0.73	0.63	0.67	0.79	0.51
0.85	0.96	2.42	0.12	-	0.21	0.23	0.24	0.37	0.36	0.61	0.63	0.67	0.60	0.68	0.72	0.61	0.65	0.77	0.49
0.66	0.68	2.06	0.16	0.23	-	0.10	0.05	0.21	0.13	0.40	0.41	0.48	0.40	0.47	0.52	0.44	0.45	0.53	0.31
0.77	0.79	2.26	0.21	0.30	0.29	-	0.12	0.27	0.21	0.51	0.53	0.56	0.48	0.52	0.54	0.47	0.49	0.59	0.37
0.66	0.70	1.78	0.27	0.34	0.16	0.33	-	0.15	0.10	0.36	0.36	0.41	0.34	0.38	0.42	0.35	0.35	0.44	0.23
0.68	0.79	2.25	0.31	0.45	0.24	0.48	0.25	-	0.18	0.48	0.49	0.56	0.47	0.50	0.52	0.47	0.43	0.58	0.35
0.48	0.53	2.56	0.31	0.42	0.20	0.35	0.18	0.22	-	0.45	0.44	0.51	0.44	0.51	0.54	0.48	0.47	0.57	0.36
0.95	1.05	0.81	1.43	1.32	0.75	1.93	0.73	1.00	0.93	-	0.08	0.08	0.08	0.41	0.50	0.42	0.44	0.43	0.33
0.70	0.85	0.72	1.31	1.19	0.72	1.56	0.70	0.91	0.77	0.09	-	0.15	0.07	0.42	0.51	0.42	0.44	0.44	0.34
1.07	1.22	0.68	1.86	1.70	1.25	1.74	1.05	1.54	1.25	0.15	0.14	-	0.07	0.44	0.54	0.46	0.49	0.48	0.35
1.03	1.21	0.64	2.13	1.67	1.41	1.91	1.12	1.66	1.33	0.23	0.16	0.12	-	0.33	0.41	0.33	0.37	0.34	0.29
1.04	1.34	0.87	1.38	1.31	1.15	1.11	0.95	1.05	1.15	0.66	0.51	0.48	0.48	-	0.08	0.08	0.10	**0.08**	0.16
1.06	1.36	1.01	1.28	1.43	1.11	1.02	0.91	0.82	1.06	0.80	0.61	0.61	0.53	0.09	-	0.10	0.09	0.32	0.28
0.99	1.30	0.89	1.07	1.11	0.98	0.91	0.80	0.91	1.02	0.73	0.53	0.58	0.49	0.09	0.08	-	**0.06**	0.18	0.21
1.05	1.36	1.06	1.46	1.30	1.28	1.16	0.93	0.97	1.12	0.88	0.66	0.72	0.60	0.16	0.15	0.09	-	0.23	0.19
1.14	1.46	0.81	1.70	1.59	1.36	1.46	1.03	1.28	1.34	0.58	0.47	0.45	0.41	0.13	0.18	0.12	0.11	-	0.23
0.93	1.11	1.17	1.07	1.02	0.91	0.94	0.66	0.89	0.89	0.76	0.64	0.59	0.57	0.28	0.36	0.26	0.19	0.18	-
