# Supplementary material for: Phylogeography of the Italian vairone (Telestes muticellus, Bonaparte 1837) inferred by microsatellite markers: evolutionary history of a freshwater fish species with a restricted and fragmented distribution
Source: BMC Evol Biol. 2010 Apr 27;10:111. doi: 10.1186/1471-2148-10-111 (PMC2868840; doi:10.1186/1471-2148-10-111)
Supplement: Additional file 3 — Allele frequencies. [file 1471-2148-10-111-S3.PDF]

Additional File 3: Allele frequencies

Population	Lsou05		Lsou08		Lsou19		Lsou10		Lsou34		Lsou09		Lsou11		Lsou21	
	allele size	frequence	allele size	frequence	allele size	frequence	allele size	frequence	allele size	frequence	allele size	frequence	allele size	frequence	allele size	frequence
Total	174	0.001	180	0.033	162	0.002	254	0.003	226	0.298	101	0.001	255	0.003	279	0.025
	178	0.003	182	0.031	174	0.067	262	0.541	228	0.176	103	0.012	257	0.005	281	0.031
	180	0.017	184	0.007	178	0.002	264	0.036	230	0.376	107	0.007	259	0.003	283	0.081
	182	0.167	186	0.018	180	0.792	266	0.006	232	0.092	109	0.033	261	0.059	285	0.121
	184	0.292	188	0.013	182	0.107	274	0.117	236	0.016	111	0.067	263	0.016	287	0.022
	186	0.026	190	0.376	184	0.004	276	0.021	238	0.014	113	0.512	265	0.012	289	0.067
	188	0.248	192	0.122	206	0.005	278	0.050	240	0.010	115	0.140	267	0.011	291	0.295
	190	0.076	194	0.201	214	0.004	280	0.223	258	0.003	117	0.052	269	0.017	293	0.209
	192	0.038	196	0.052	216	0.018	282	0.001	266	0.012	119	0.042	271	0.045	295	0.072
	194	0.087	198	0.026			290	0.003	268	0.003	121	0.047	273	0.008	297	0.071
	196	0.005	200	0.049							123	0.046	275	0.033	299	0.003
	198	0.019	202	0.033							125	0.020	277	0.663	303	0.004
	200	0.010	204	0.017							127	0.020	279	0.116		
	202	0.001	206	0.009							129	0.001	281	0.005		
	204	0.009	208	0.007							135	0.003	283	0.002		
	206	0.001	210	0.004									285	0.002		
	212	0.001	212	0.002									291	0.002		
1	180	0.071	182	0.036	180	0.679	262	0.393	226	0.429	109	0.045	261	0.045	285	0.143
	182	0.107	190	0.393	182	0.321	264	0.214	228	0.214	113	0.500	277	0.909	287	0.071
	184	0.429	192	0.214			266	0.036	230	0.321	117	0.091	279	0.045	291	0.071
	186	0.143	194	0.107			274	0.250	232	0.036	119	0.182			293	0.286
	188	0.071	200	0.179			280	0.071			121	0.091			295	0.357
	192	0.036	210	0.071			282	0.036			135	0.091			299	0.071
	194	0.036														
	198	0.107														
2	184	0.575	182	0.125	180	0.900	262	0.625	226	0.361	109	0.050	271	0.063	279	0.050
	188	0.250	190	0.450	182	0.100	274	0.375	228	0.306	111	0.025	277	0.813	281	0.050
	192	0.050	192	0.350					230	0.306	113	0.775	279	0.125	283	0.050
	204	0.125	194	0.050					236	0.028	115	0.050			285	0.050
			196	0.025							119	0.100			287	0.150
															289	0.200
															291	0.350
															293	0.100
3	180	0.375	186	0.188	180	0.500	262	0.875	226	0.313	103	0.250	271	0.167	285	0.125
	182	0.125	188	0.063	182	0.500	264	0.125	230	0.250	111	0.125	277	0.583	287	0.063
	188	0.250	190	0.375					232	0.125	113	0.500	279	0.250	293	0.188
	190	0.125	192	0.125					238	0.313	115	0.063			295	0.625
	192	0.125	200	0.188							119	0.063				
			202	0.063												

Population	Lsou05		Lsou08		Lsou19		Lsou10		Lsou34		Lsou09		Lsou11		Lsou21	
	allele size	frequence	allele size	frequence	allele size	frequence	allele size	frequence	allele size	frequence	allele size	frequence	allele size	frequence	allele size	frequence
4	174	0.125	190	0.625	180	0.500	262	1.000	230	0.875	113	1.000	271	0.125	285	0.125
	180	0.125	192	0.250	182	0.500			232	0.125			277	0.750	291	0.125
	184	0.500	196	0.125									279	0.125	293	0.500
	188	0.125													295	0.250
	206	0.125														
5	184	0.500	186	0.042	180	0.833	262	0.818	226	0.417	109	0.300	271	0.208	283	0.083
	188	0.292	190	0.917	182	0.167	274	0.182	228	0.125	113	0.600	277	0.792	285	0.417
	190	0.042	194	0.042					230	0.208	115	0.100			291	0.125
	194	0.083							232	0.250					293	0.292
	196	0.083													295	0.042
															297	0.042
6	184	0.458	182	0.042	180	0.792	262	0.792	226	0.250	113	0.636	271	0.091	279	0.083
	188	0.458	186	0.042	182	0.208	264	0.042	228	0.250	115	0.136	277	0.864	281	0.125
	194	0.083	188	0.042			274	0.167	230	0.250	117	0.227	279	0.045	287	0.083
			190	0.625					232	0.042					289	0.083
			194	0.208					236	0.208					291	0.167
			196	0.042											293	0.042
															295	0.083
															297	0.333
7	180	0.033	182	0.067	180	0.867	262	0.600	226	0.500	107	0.042	277	0.750	281	0.033
	184	0.500	190	0.600	182	0.133	274	0.333	228	0.267	113	0.792	279	0.250	283	0.267
	188	0.100	192	0.167			276	0.067	230	0.133	117	0.125			285	0.100
	190	0.133	194	0.100					232	0.033	127	0.042			289	0.033
	192	0.100	196	0.067					236	0.033					291	0.233
	194	0.100							240	0.033					293	0.133
	204	0.033													295	0.033
															297	0.167
8	184	0.367	182	0.033	180	0.933	262	0.833	226	0.133	113	0.750	277	0.875	279	0.033
	186	0.033	186	0.100	182	0.067	274	0.167	228	0.100	115	0.179	279	0.125	281	0.100
	188	0.433	190	0.533					230	0.433	121	0.071			283	0.033
	194	0.167	192	0.300					232	0.267					285	0.167
			194	0.033					236	0.067					289	0.033
															291	0.067
															293	0.433
															295	0.033
															297	0.100

Population	Lsou05		Lsou08		Lsou19		Lsou10		Lsou34		Lsou09		Lsou11		Lsou21	
	allele size	frequence	allele size	frequence	allele size	frequence	allele size	frequence	allele size	frequence	allele size	frequence	allele size	frequence	allele size	frequence
9	178	0.033	182	0.067	180	0.800	262	0.867	226	0.633	111	0.042	277	1.000	279	0.133
	184	0.367	186	0.033	182	0.200	274	0.133	228	0.067	113	0.625			285	0.167
	188	0.300	190	0.600					230	0.200	115	0.083			287	0.033
	190	0.067	192	0.133					232	0.100	117	0.167			291	0.067
	192	0.133	194	0.033							121	0.083			293	0.467
	194	0.033	196	0.033											295	0.100
	198	0.067	200	0.067											297	0.033
			202	0.033												
10	184	0.433	182	0.033	180	0.900	262	0.536	226	0.267	111	0.045	277	1.000	279	0.067
	186	0.100	186	0.033	182	0.100	264	0.107	228	0.133	113	0.727			285	0.233
	188	0.133	188	0.033			274	0.286	230	0.333	115	0.136			291	0.133
	190	0.033	190	0.533			276	0.071	232	0.267	119	0.091			293	0.267
	192	0.167	192	0.100											295	0.133
	194	0.033	194	0.033											297	0.167
	198	0.100	196	0.033												
			198	0.067												
			200	0.100												
			202	0.033												
11	180	0.033	182	0.067	180	0.767	262	0.833	226	0.267	113	0.727	257	0.042	279	0.033
	184	0.367	186	0.100	182	0.233	264	0.067	228	0.033	115	0.136	277	0.958	281	0.033
	186	0.100	190	0.533			274	0.067	230	0.500	119	0.091			283	0.033
	188	0.267	192	0.167			276	0.033	232	0.200	121	0.045			285	0.067
	190	0.100	196	0.033											287	0.067
	192	0.067	200	0.067											293	0.533
	198	0.033	204	0.033											295	0.233
	200	0.033														
12	184	0.318	182	0.182	180	0.818	262	0.545	226	0.318	113	0.350	271	0.214	279	0.045
	186	0.045	188	0.136	182	0.182	264	0.182	228	0.318	115	0.050	277	0.786	285	0.227
	188	0.227	190	0.591			274	0.045	230	0.227	119	0.050			293	0.545
	190	0.045	192	0.091			276	0.227	232	0.136	121	0.350			295	0.045
	192	0.227									123	0.100			297	0.136
	194	0.091									125	0.100				
	198	0.045														
13	178	0.038	182	0.077	180	0.731	262	0.769	226	0.154	107	0.100	271	0.100	279	0.192
	184	0.538	190	0.846	182	0.269	264	0.038	228	0.077	111	0.100	277	0.900	281	0.038
	186	0.077	194	0.038			274	0.115	230	0.538	113	0.500			285	0.077
	188	0.154	200	0.038			276	0.077	232	0.192	115	0.100			289	0.038
	190	0.077							236	0.038	119	0.100			291	0.077
	192	0.038									125	0.100			293	0.385

Population	Lsou05		Lsou08		Lsou19		Lsou10		Lsou34		Lsou09		Lsou11		Lsou21	
	allele size	frequence	allele size	frequence	allele size	frequence	allele size	frequence	allele size	frequence	allele size	frequence	allele size	frequence	allele size	frequence
13	194	0.077													295	0.115
															297	0.038
															303	0.038
14	184	0.200	182	0.033	180	0.567	262	0.643	226	0.133	113	0.750	255	0.042	279	0.167
	188	0.467	184	0.067	182	0.433	264	0.321	228	0.067	115	0.050	263	0.042	281	0.033
	192	0.100	190	0.467			276	0.036	230	0.367	119	0.200	271	0.042	285	0.267
	194	0.033	192	0.233					232	0.433			277	0.875	287	0.033
	198	0.200	194	0.100											289	0.100
			196	0.033											291	0.033
			198	0.033											293	0.267
			200	0.033											295	0.100
15	178	0.033	182	0.033	180	1.000	262	0.625	226	0.100	111	0.091	261	0.063	279	0.033
	184	0.467	186	0.033			264	0.042	228	0.033	113	0.636	269	0.063	283	0.033
	186	0.033	188	0.033			274	0.208	230	0.733	115	0.136	277	0.875	285	0.100
	188	0.333	190	0.333			290	0.125	232	0.133	117	0.045			287	0.033
	190	0.067	192	0.100							121	0.045			291	0.367
	192	0.033	194	0.100							123	0.045			293	0.100
	198	0.033	198	0.033											295	0.033
			202	0.300											297	0.300
			210	0.033												
16	180	0.042	186	0.042	180	0.958	262	0.625	226	0.167	109	0.200	275	0.167	283	0.045
	184	0.375	190	0.708	182	0.042	266	0.083	230	0.833	111	0.050	277	0.833	285	0.136
	188	0.458	192	0.125			274	0.250			113	0.450			291	0.091
	190	0.042	196	0.083			276	0.042			115	0.050			293	0.500
	194	0.042	200	0.042							119	0.150			295	0.045
	198	0.042									121	0.100			297	0.045
															303	0.136
17	184	0.167	190	0.433	180	0.800	254	0.100	226	0.367	109	0.036	275	0.091	281	0.036
	186	0.067	192	0.233	182	0.200	262	0.667	230	0.400	111	0.071	277	0.909	283	0.107
	188	0.400	194	0.167			266	0.033	232	0.100	113	0.250			285	0.214
	190	0.033	206	0.067			274	0.200	236	0.100	115	0.500			293	0.429
	192	0.067	208	0.067					240	0.033	117	0.071			295	0.071
	194	0.167	212	0.033							123	0.071			297	0.143
	198	0.033														
	202	0.033														
	212	0.033														

Population	Lsou05		Lsou08		Lsou19		Lsou10		Lsou34		Lsou09		Lsou11		Lsou21	
	allele size	frequence	allele size	frequence	allele size	frequence	allele size	frequence	allele size	frequence	allele size	frequence	allele size	frequence	allele size	frequence
18	184	0.700	190	0.200	180	1.000	262	0.833	230	0.700	109	0.107	275	0.286	279	0.067
	188	0.200	192	0.067			274	0.167	232	0.033	113	0.536	277	0.643	281	0.200
	196	0.100	194	0.367					240	0.267	115	0.357	279	0.071	283	0.133
			196	0.033											285	0.200
			204	0.133											291	0.133
			206	0.167											293	0.100
			210	0.033											297	0.167
19	180	0.067	190	0.433	180	0.567	262	0.800	226	0.233	113	1.000	275	0.100	285	0.533
	182	0.033	192	0.333	182	0.333	264	0.200	230	0.767			277	0.800	289	0.200
	184	0.133	194	0.133	184	0.100							279	0.100	291	0.033
	188	0.633	196	0.067											293	0.133
	190	0.100	200	0.033											295	0.067
	192	0.033													297	0.033
20	182	0.150	180	0.850	180	1.000	262	0.700	226	0.800					287	0.150
	184	0.300	194	0.100			280	0.300	230	0.050					289	0.750
	188	0.450	212	0.050					232	0.100					291	0.100
	190	0.100							236	0.050						
21	182	0.050	180	0.900	180	0.800	262	0.722	226	0.833					289	0.850
	184	0.200	194	0.050	182	0.200	274	0.111	230	0.056					291	0.150
	188	0.700	196	0.050			280	0.167	232	0.111						
	198	0.050														
22	190	0.733	194	0.333	180	0.067	280	1.000	236	0.067	113	0.200			291	1.000
	194	0.267	196	0.333	206	0.167			238	0.333	127	0.800				
			198	0.100	214	0.133			258	0.100						
			202	0.100	216	0.633			266	0.400						
			204	0.133					268	0.100						
23	184	0.100	190	1.000	180	1.000	262	1.000	230	1.000	109	0.100	255	0.167	281	0.233
	188	0.233									111	0.100	275	0.167	283	0.567
	194	0.467									113	0.700	277	0.667	285	0.067
	200	0.200									121	0.100			297	0.133
24	184	0.300	190	0.933	180	1.000	262	1.000	226	0.067	111	0.154	261	0.222	281	0.033
	188	0.133	192	0.067					230	0.900	113	0.077	263	0.111	283	0.700
	194	0.567							232	0.033	117	0.077	275	0.111	287	0.067
											123	0.692	277	0.556	291	0.200

Population	Lsou05		Lsou08		Lsou19		Lsou10		Lsou34		Lsou09		Lsou11		Lsou21	
	allele size	frequence	allele size	frequence	allele size	frequence	allele size	frequence	allele size	frequence	allele size	frequence	allele size	frequence	allele size	frequence
25	180	0.133	184	0.133	178	0.067	262	0.767	226	0.333	111	0.083	265	0.091	281	0.067
	184	0.167	190	0.467	180	0.933	266	0.033	230	0.633	113	0.667	277	0.909	283	0.233
	188	0.567	192	0.067			274	0.200	236	0.033	123	0.250			285	0.200
	192	0.067	200	0.300											291	0.333
	194	0.067	206	0.033											295	0.167
26	184	0.033	182	0.267	180	0.900	262	0.733	228	0.167	111	0.222			281	0.100
	188	0.567	190	0.433	182	0.100	274	0.267	230	0.833	113	0.389			283	0.533
	190	0.367	192	0.300							121	0.389			285	0.233
	194	0.033													293	0.133
27	184	0.200	188	0.100	180	0.933	262	0.567	226	0.267	113	0.400	271	0.167	285	0.167
	186	0.033	190	0.300	182	0.067	266	0.033	228	0.100	115	0.533	275	0.167	291	0.367
	188	0.333	192	0.300			274	0.267	230	0.567	123	0.033	277	0.583	293	0.267
	192	0.033	194	0.033			276	0.133	232	0.067	127	0.033	285	0.083	295	0.167
	194	0.200	196	0.033											297	0.033
	200	0.100	204	0.133												
	204	0.100	206	0.033												
			208	0.067												
28	182	0.033	190	0.200	180	1.000	262	0.933	226	0.100	113	0.900	263	0.100	285	0.333
	184	0.700	192	0.033			276	0.067	230	0.467	115	0.067	273	0.050	289	0.167
	186	0.033	194	0.333					232	0.433	117	0.033	275	0.100	293	0.433
	188	0.067	196	0.333									277	0.750	297	0.067
	192	0.167	198	0.100												
29	182	0.033	188	0.067	180	1.000	262	0.800	226	0.433	111	0.083	261	0.036	285	0.167
	184	0.433	190	0.200			264	0.067	230	0.467	113	0.500	263	0.071	289	0.100
	188	0.500	192	0.400			274	0.067	232	0.100	115	0.167	271	0.250	291	0.033
	194	0.033	196	0.100			280	0.067			117	0.125	275	0.071	293	0.033
			198	0.033							119	0.083	277	0.536	297	0.667
			200	0.067							125	0.042	279	0.036		
			202	0.033												
			208	0.100												
30	182	0.667	190	0.033	174	0.600	262	0.167	226	0.900	101	0.038	261	0.182	283	0.033
	184	0.200	194	0.400	180	0.233	274	0.100	232	0.100	109	0.038	277	0.727	287	0.033
	190	0.067	196	0.067	182	0.167	276	0.067			113	0.269	279	0.045	291	0.933
	194	0.067	200	0.433			278	0.033			115	0.423	283	0.045		
			202	0.067			280	0.633			119	0.038				
											121	0.077				
											123	0.038				
											125	0.038				
											127	0.038				

Population	Lsou05		Lsou08		Lsou19		Lsou10		Lsou34		Lsou09		Lsou11		Lsou21	
	allele size	frequence	allele size	frequence	allele size	frequence	allele size	frequence	allele size	frequence	allele size	frequence	allele size	frequence	allele size	frequence
31	182	0.533	184	0.033	174	0.367	262	0.067	226	1.000	103	0.042	259	0.038	285	0.033
	184	0.333	190	0.033	180	0.633	278	0.067			109	0.042	261	0.269	291	0.967
	186	0.100	194	0.233			280	0.867			111	0.125	265	0.077		
	194	0.033	196	0.067							113	0.333	269	0.077		
			198	0.133							115	0.208	277	0.462		
			200	0.167							119	0.042	279	0.077		
			202	0.333							121	0.083				
											123	0.042				
											125	0.083				
32	182	0.733	192	0.300	174	0.767	278	0.200	226	0.833	103	0.045	261	0.313	291	1.000
	190	0.100	194	0.533	180	0.233	280	0.800	230	0.167	109	0.045	267	0.063		
	194	0.167	196	0.067							111	0.136	269	0.063		
			202	0.100							113	0.318	271	0.063		
											115	0.182	273	0.188		
											121	0.182	277	0.063		
											125	0.091	279	0.188		
													281	0.063		
33	182	0.600	188	0.067	162	0.067	278	0.100	226	0.667	103	0.083	261	0.500	291	0.900
	184	0.067	194	0.200	174	0.600	280	0.900	228	0.200	107	0.042	269	0.125	293	0.100
	186	0.133	196	0.133	180	0.333			230	0.133	109	0.083	279	0.250		
	190	0.167	198	0.267							113	0.083	281	0.125		
	194	0.033	200	0.033							115	0.208				
			202	0.133							117	0.458				
			204	0.167							119	0.042				
34	182	0.900	190	0.050	180	0.950	278	0.200	228	0.900	109	0.071	257	0.200	287	0.050
	188	0.100	194	0.850	184	0.050	280	0.800	230	0.100	111	0.286	261	0.200	291	0.550
			196	0.100							113	0.214	263	0.100	293	0.250
											119	0.071	267	0.300	295	0.150
											121	0.071	271	0.100		
											123	0.071	279	0.100		
											125	0.071				
											127	0.143				
35	182	0.727	182	0.045	180	1.000	278	0.091	228	1.000	109	0.063	261	0.333	291	0.273
	188	0.182	190	0.045			280	0.909			111	0.125	267	0.083	293	0.727
	190	0.091	194	0.818							113	0.625	269	0.167		
			196	0.091							117	0.063	273	0.083		
											123	0.063	279	0.333		
											129	0.063				

Population	Lsou05		Lsou08		Lsou19		Lsou10		Lsou34		Lsou09		Lsou11		Lsou21	
	allele size	frequence	allele size	frequence	allele size	frequence	allele size	frequence	allele size	frequence	allele size	frequence	allele size	frequence	allele size	frequence
36	182	0.633	186	0.100	180	1.000	278	0.233	228	1.000	107	0.036	259	0.042	289	0.067
	184	0.067	190	0.400			280	0.767			109	0.036	261	0.042	291	0.533
	188	0.033	194	0.500							111	0.214	263	0.083	293	0.400
	190	0.267									113	0.357	265	0.083		
											115	0.071	267	0.083		
											117	0.036	269	0.125		
											119	0.107	277	0.042		
											121	0.071	279	0.458		
											123	0.036	291	0.042		
											125	0.036				
37	182	0.450	186	0.050	180	1.000	278	0.300	228	1.000	109	0.071	265	0.167	291	0.450
	184	0.450	190	0.150			280	0.700			111	0.429	279	0.833	293	0.550
	188	0.050	194	0.800							115	0.143				
	190	0.050									117	0.143				
											125	0.071				
											127	0.143				
38	182	0.875	194	1.000	180	1.000	280	1.000	228	1.000	103	0.167	279	1.000	291	1.000
	184	0.125									109	0.167				
											111	0.167				
											113	0.167				
											115	0.333				
39	182	0.500	194	0.778	180	0.972	274	0.194	226	0.056	111	0.125	275	0.167	289	0.167
	184	0.139	196	0.056	182	0.028	278	0.556	228	0.417	113	0.125	279	0.833	291	0.583
	186	0.028	198	0.111			280	0.250	230	0.417	115	0.250			295	0.222
	188	0.111	200	0.056					232	0.111	117	0.250			299	0.028
	190	0.028									125	0.250				
	194	0.194														
