# Supplementary figures and images for: Phylogeography of the Italian vairone (Telestes muticellus, Bonaparte 1837) inferred by microsatellite markers: evolutionary history of a freshwater fish species with a restricted and fragmented distribution
Source: BMC Evol Biol. 2010 Apr 27;10:111. doi: 10.1186/1471-2148-10-111 (PMC2868840; doi:10.1186/1471-2148-10-111)

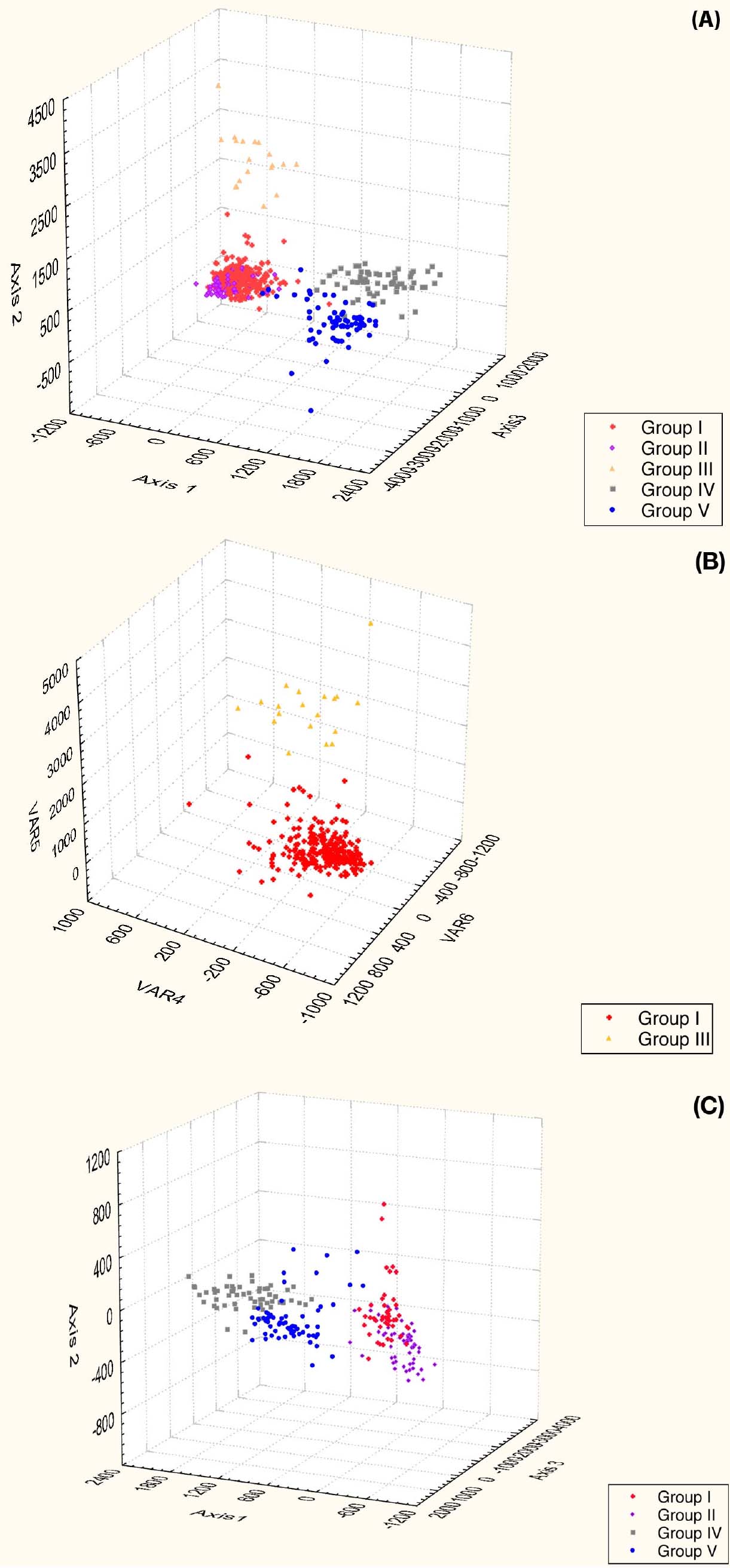

Supplement: Additional file 4 — Supplemental Figure. [file 1471-2148-10-111-S4.JPEG]

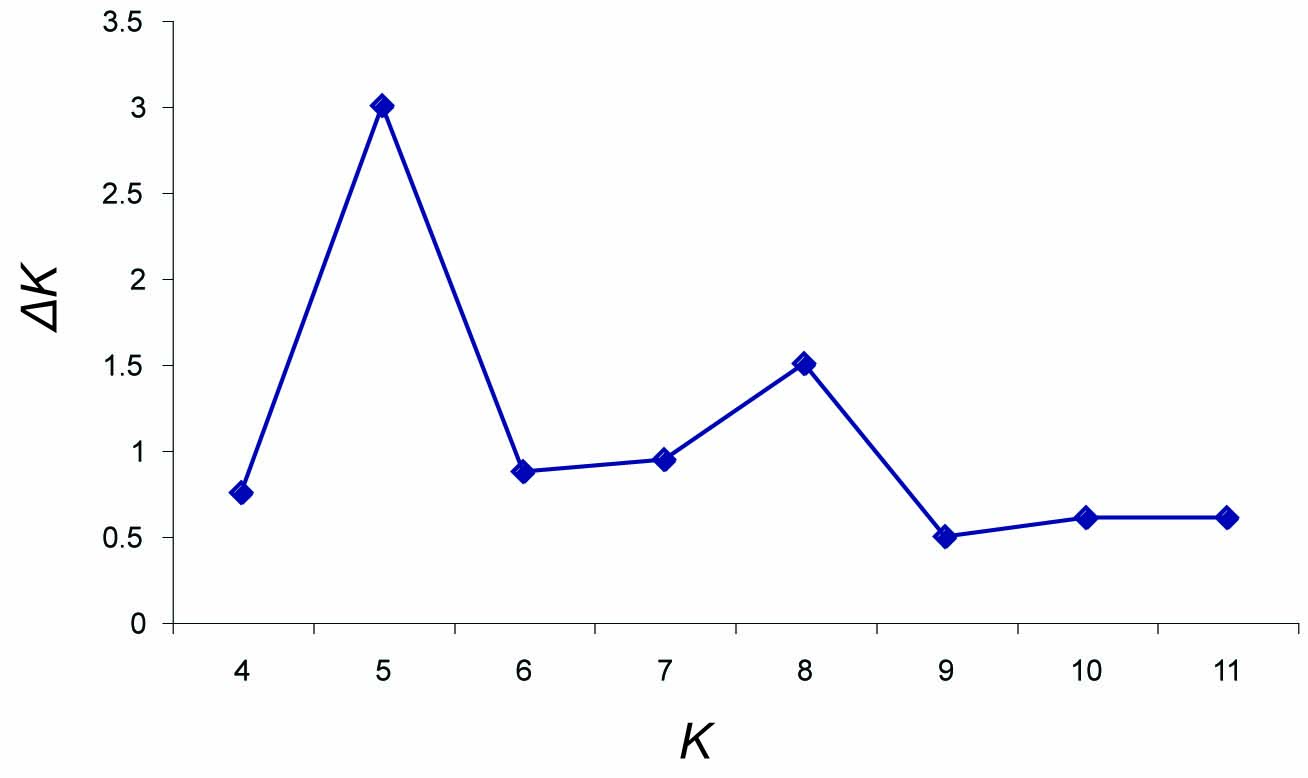

Supplement: Additional file 5 — Supplemental Figure. [file 1471-2148-10-111-S5.JPEG]
