# Supplementary material for: Phylogeography of the Italian vairone (Telestes muticellus, Bonaparte 1837) inferred by microsatellite markers: evolutionary history of a freshwater fish species with a restricted and fragmented distribution
Source: BMC Evol Biol. 2010 Apr 27;10:111. doi: 10.1186/1471-2148-10-111 (PMC2868840; doi:10.1186/1471-2148-10-111)
Supplement: Additional file 6 — AMOVA results. [file 1471-2148-10-111-S6.PDF]

Additional File 6: AMOVA results

	Variance component	P value	Fixation indexes	% of variation
Among groups	Va = 0.67919	< 0.0001	Φct = 0.29499	29.5
Among populations within groups	Vb = 0.17737	< 0.0001	Φsc = 0.10927	7,7
Within populations	Vc = 1.44583	< 0.0001	Φst = 0.372.3	62.8
