# Supplementary material for: Phylogeography of the Italian vairone (Telestes muticellus, Bonaparte 1837) inferred by microsatellite markers: evolutionary history of a freshwater fish species with a restricted and fragmented distribution
Source: BMC Evol Biol. 2010 Apr 27;10:111. doi: 10.1186/1471-2148-10-111 (PMC2868840; doi:10.1186/1471-2148-10-111)
Supplement: Additional file 7 — FIS values. [file 1471-2148-10-111-S7.PDF]

Additional file 7: FIS values

Pop.	FIS	P
01	0.07801	0.171065
02	-0.08528	0.933529
03	0	0.527859
04	0.17808	0.191593
05	0.01449	0.502444
06	-0.11302	0.947214
07	0.16309	0.008798
08	0.07557	0.198436
09	0.00435	0.516129
10	-0.03444	0.717498
11	0.08571	0.084066
12	0.04875	0.322581
13	0.08683	0.166178
14	0.02742	0.403715
15	0.02477	0.434018
16	-0.11236	0.931574
17	0.08139	0.148583
18	0.04355	0.335288
19	-0.00957	0.597263
20	0.05736	0.368524
21	0.11561	0.206256
23	0.25134	0.02346
24	-0.00685	0.581623
25	0.0469	0.337243
26	-0.06207	0.743891
27	0.00191	0.5826
28	-0.025	0.676442
29	0.04967	0.312805
30	0.15152	0.055718
31	0.13248	0.107527
32	0.328	0
33	0.05514	0.273705
34	0.4375	0
35	0.28105	0.016618
36	0.17179	0.084066
37	0.12821	0.253177
38	0	1
39	0.00109	0.461388
